# Binary-FRET reveals transient excited-state structure associated with activity-dependent CaMKII - NR2B binding and adaptation

**DOI:** 10.1038/s41467-022-33795-8

**Published:** 2022-10-25

**Authors:** Tuan A. Nguyen, Henry L. Puhl, Kirk Hines, Daniel J. Liput, Steven S. Vogel

**Affiliations:** 1 Laboratory of Biophotonics and Quantum Biology, NIAAA, NIH, Bethesda, USA; 2 Laboratory for Integrative Neuroscience, NIAAA, NIH, Bethesda, USA

**Keywords:** Molecular biophysics, Biological fluorescence, Biophysical methods, Synaptic plasticity

## Abstract

Synaptic functions are mediated and modulated by a coordinated choreography of protein conformational changes and interactions in response to intracellular calcium dynamics. Time-lapse Förster resonance energy transfer can be used to study the dynamics of both conformational changes and protein-protein interactions simultaneously under physiological conditions if two resonance energy transfer reactions can be multiplexed. Binary-FRET is a technique developed to independently monitor the dynamics of calcium-calmodulin dependent protein kinase-II catalytic-domain pair separation in the holoenzyme, and its role in establishing activity-dependent holoenzyme affinity for the NR2B binding fragment of the N-methyl-D-aspartate receptor. Here we show that a transient excited-state intermediate exists where paired catalytic-domains in the holoenzyme first separate prior to subsequent NR2B association. Additionally, at non-saturating free calcium concentrations, our multiplexed approach reveals that the holoenzyme exhibits a biochemical form of plasticity, calcium dependent adaptation of T-site ligand binding affinity.

## Abstract (web summary)

FRET can be used to study conformational changes and protein-protein interactions. Here the authors report Binary-FRET for monitoring two FRET reactions, one encoded in the fluorescence lifetime of the donor, another encoded in its anisotropy, and monitor the dynamics of CaMKII and its interaction with NR2B.

## Introduction

The calcium-calmodulin-dependent protein kinase-II (CaMKII) is thought to play an important role in regulating synaptic efficacy, learning, and memory^1,2^. CaMKII is activated by elevated calcium and can then translocate from dendrites into post-synaptic spines^3–7^ in response to prior neuronal activity^2,8^. Synaptic transmission causes calcium influx through NMDA receptors in post-synaptic densities (PSDs) when glutamate is released by presynaptic terminals and when postsynaptic terminals depolarize^9,10^. These coordinated activities can be thought of as a trigger to initiate an eligibility trace, a transient memory mechanism that flags a period of eligibility when past experiences can influence future actions^11,12^ to enable synapse-specific reinforcement learning. NMDA receptor gating creates transient microdomains and gradients of elevated calcium which CaMKII might sense. Long-term potentiation (LTP) is a synapse-based model for memory that has an early stage that is dependent on post-translational modifications and lasts for a few hours^13,14^. Knock-out experiments have implicated CaMKIIα as a key component of LTP^15,16^. Thus, CaMKII is a good candidate for being a constituent of a biological eligibility trace^17^ which might involve structural changes in the holoenzyme that lasts for hours. Holoenzyme translocation is thought to be mediated by its binding to the NR2B subunit of the N-methyl-d-aspartate (NMDA) receptors in PSDs^4,5,18,19^. It is unclear how stable CaMKII–NMDA receptor interactions are^6,17,20^ or if a specific holoenzyme conformation is associated with binding. CaMKII forms a holoenzyme composed of 8–14 subunits arranged as catalytic domain pairs distributed around a central oligomerization core domain^21,22^. The existence and potential importance of catalytic domain pairing for kinase activation have only recently been appreciated following crystallographic^23^ and affinity studies^24^ using isolated catalytic domains, as well as from Förster resonance energy transfer (FRET)^21,25–27^ and electron microscopy^28^ studies using the holoenzyme. Each subunit catalytic domain has a T-site (the targeted binding site for NR2B). In hippocampal neurons, calcium influx triggered a conformational change consistent with catalytic domain pair separation^25^. It is not known if activation triggers catalytic domain pair separation, or alternatively if T-site ligands trigger unpairing^27^. Surprisingly, monomeric mutants of CaMKII did not translocate to spines^18,29^, suggesting that some emergent feature of the holoenzyme structure facilitates translocation. Based on the extensive CaMKII experimental literature^2,30^, and these recent studies revealing catalytic domain pairing, we hypothesize that autoinhibited CaMKII holoenzyme catalytic domain pairing conceals T-sites, thus preventing NMDA receptor binding. We postulate that when calcium-calmodulin (CaM) binds it triggers a conformational change where catalytic domain pairs extend from the core domain and separate to allow access to these concealed binding sites. If true, we predict that, 1. Catalytic-domain pair separation should occur upon activation, even in the absence of a T-site ligand, 2. Unpairing should precede ligand binding, and 3. T-site ligand binding should only occur when catalytic domain pairs separate. Furthermore, if CaMKII catalytic-domain separation is a component of an eligibility trace, we expect activation-triggered unpairing will have a transient duration on a time scale that can persist for hours.

FRET involves the incoherent transfer of excited-state energy from a fluorophore to an acceptor chromophore via near-field coulombic dipole–dipole coupling^31–34^. FRET occurs when three conditions are met: proximity of donor and acceptor chromophores (~1–10 nm), permissive dipole orientations, and spectral overlap of donor emission and acceptor absorption. As a research tool, FRET microscopy is used to monitor the interactions of either intrinsically fluorescent molecules, or molecules tagged, often genetically, with a fluorescent protein. FRET’s dependence on both proximity and dipole orientation is the foundation for investigating protein conformational changes and protein–protein interactions. Potentially, if two FRET reactions could be monitored simultaneously and independently, our T-site accessibility model could be directly tested. Following two FRET reactions independently under physiological conditions, however, is problematic because fluorescent protein (FP) donor and acceptor pairs typically have broad and overlapping excitation and emission spectra.

Here, we show that the fluorescent emission of a single FP can be used to monitor two FRET signals. Specifically, a homo-FRET signal can be encoded in the emission polarization, and an independent hetero-FRET signal can be encoded in its fluorescence lifetime. We call this approach for monitoring two multiplexed FRET reactions Binary-FRET.

## Results

### Binary-FRET multiplexing theory

**Fig. 1:**
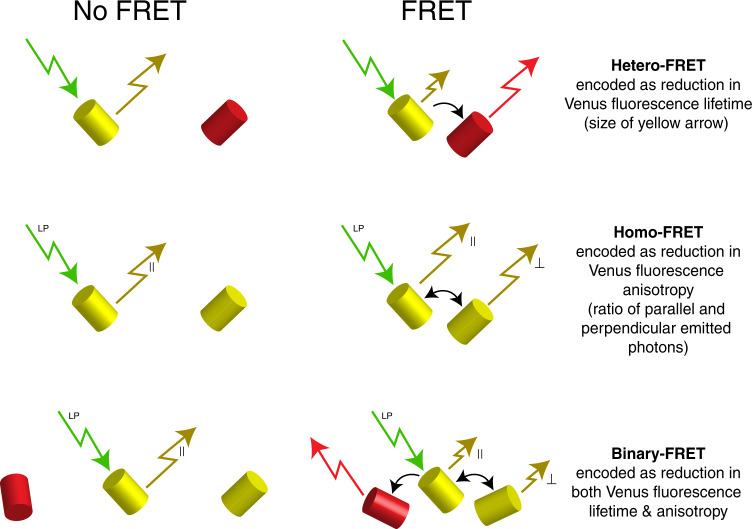
The theoretical basis for binary-FRET: hetero-FRET and homo-FRET multiplexing. The hetero-FRET donor lifetime change is indicated by the size of the yellow emission arrow, while the homo-FRET change in emission anisotropy is indicated by the ratio of yellow parallel and perpendicular components.

There are two categories of FRET, hetero-FRET, where FRET donors and acceptors are spectrally distinct, and homo-FRET, where they share the same spectra (Fig. 1). Hetero-FRET and homo-FRET are both sensitive to the separation between donors and acceptors and to the dipole orientation factor^35,36^. Thus, both can be used to study protein–protein interactions and/or protein conformational changes. Hetero-FRET is encoded in the decay constant of the FRET donor’s *fluorescence lifetime* (*τ*)^37^, while homo-FRET is encoded in the *fluorescence polarization correlation time* (*θ*) decay constant^21,38–42^. When a fluorophore is excited with a short pulse of linear polarized light its fluorescence emission decays as a function of time, *I*(*t*), and can be separated, using a polarizing beam splitter into orthogonal parallel and perpendicular decay components.

1 $${I}_{\parallel }\left(t\right)=G{F}_{0}\cdot {{{{{{\rm{e}}}}}}}^{\left(-t/\tau \right)}\left(2{r}_{0}\cdot {{{{{{\rm{e}}}}}}}^{\left(-t/\theta \right)}\right)+1$$

2 $${I}_{\perp }\left(t\right)={F}_{0}\cdot {{{{{{\rm{e}}}}}}}^{\left(-t/\tau \right)}\left({-r}_{0}\cdot {{{{{{\rm{e}}}}}}}^{\left(-t/\theta \right)}\right)+1$$

*F*_0_ is proportional to the amplitude of the donor’s fluorescence lifetime decay. The *limiting anisotropy*, *r*_0_, is the amplitude of the anisotropy decay, and *G* is a constant that accounts for differences in the sensitivity of the photon detectors. The decays of *I*_‖_(*t*) and *I*_⟂_(*t*) are functions of both *τ* and *θ* and therefore are influenced by both hetero-FRET and homo-FRET.

The hetero-FRET and homo-FRET information embedded in *τ* and *θ* can be isolated and recovered from *I*_‖_(*t*) and *I*_⟂_(*t*) decays by substituting Eqs. (1) and (2) into the equations for time-resolved anisotropy, *r*(*t*), and fluorescence lifetime, *I*(*t*),

3 $$r\left(t\right)=\frac{{I}_{\parallel }\left(t\right)-G\cdot {I}_{\perp }\left(t\right)}{{I}_{\parallel }\left(t\right)+2G\cdot {I}_{\perp }\left(t\right)}$$

4 $$I\left(t\right)={I}_{\parallel }\left(t\right)+2G\cdot {I}_{\perp }\left(t\right)$$

Substitution of Eqs. (1), (2) into Eq. (3) yields Eq. (5):

5 $$r\left(t\right)={r}_{0}\cdot {{{{{{\rm{e}}}}}}}^{-t/\theta }$$

indicating that the fluorescence anisotropy decay calculated from the parallel and perpendicular decay components is not a function of *τ*, and presumably contains only homo-FRET and molecular rotation information. Similarly, the substitution of Eqs. (1), (2) into Eq. (4) yields Eq. (6):

6 $$I\left(t\right)={F}_{0}\left(1+2G\right)\cdot {{{{{{\rm{e}}}}}}}^{-t/\tau }$$

indicating that the decay of the donor fluorescence lifetime is not a function of θ, and only contains hetero-FRET information.

### Validating binary-FRET

**Fig. 2:**
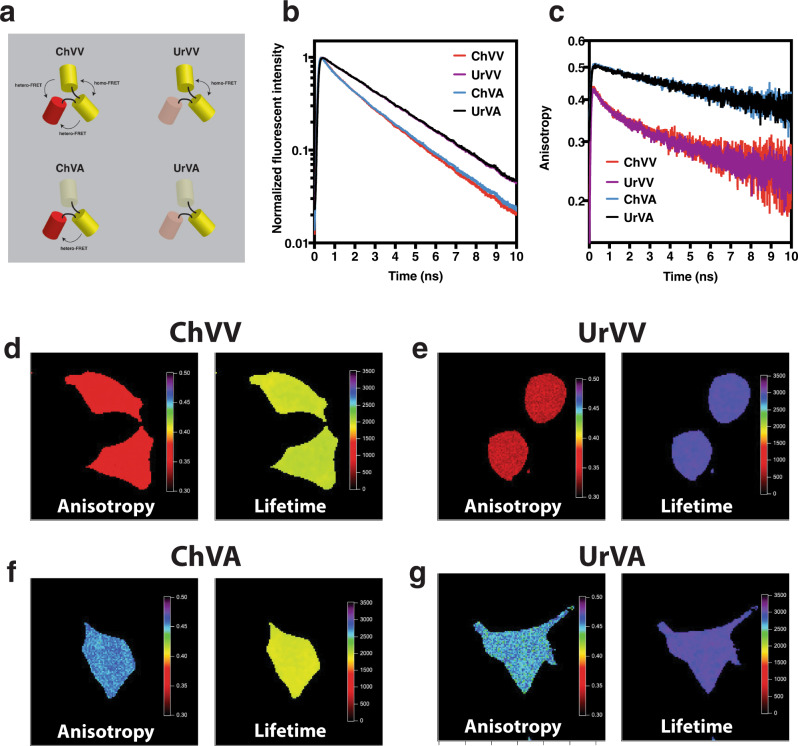
Validating binary-FRET. **a** Cartoon depicting fluorescent protein control constructs. **b** In vitro fluorescence lifetime decays, **c** In vitro time-resolved, fluorescence anisotropy decays. Steady-state anisotropy and fluorescence lifetime images (128 × 128 pixels corresponding to 42.6 µ × 42.6 µ) of HEK cells expressing ChVV (**d**), UrVV (**e**), ChVA (**f**) and UrVA (**g**). Anisotropy color bar ranged from 0.3 (dark red) to 0.5 (dark purple). Lifetime color bars are in picoseconds and ranged from 0 ps (dark red) to 3500 ps (dark purple). Source data are provided as a Source Data file.

To test if hetero-FRET and homo-FRET signals can be demodulated from parallel and perpendicular fluorescence anisotropy decay components we generated four control samples, each comprised of three concatenated fluorescent proteins using short (5–6 amino acids) linkers (Fig. 2a). The fluorescent proteins (FPs) used were monomeric yellow fluorescent protein mVenus, monomeric red fluorescent protein mCherry, and their tyrosine to cysteine (Y → C) mutants (mVenus_Y67C_, Amber and mCherry_Y72C_, Uranus). These point mutations in the tyrosine residues that form their chromophores prevent FP chromophore formation and therefore cannot participate in a FRET reaction (Supplementary Fig. 1). The first control was mCherry–mVenus–mVenus (ChVV). Since all three FPs in this construct are within 10 nm of each other, we reasoned that this sample should display both hetero-FRET (between mVenus and the mCherry) as well as homo-FRET (between the two mVenus molecules). The second control was mCherry_Y72C_–mVenus–mVenus (UrVV). We only expect homo-FRET between the two mVenus chromophores in this sample. The next control was mCherry–mVenus–mVenus_Y67C_ (ChVA). We expect to see only hetero-FRET between the mVenus donor and the mCherry acceptor in this sample. Finally, our last control was mCherry_Y72C_–mVenus–mVenus_Y67C_ (UrVA). This sample should display no homo-FRET or hetero-FRET. Note that these samples all have at least one mVenus fluorophore and binary-FRET uses its emission alone to detect homo- and hetero-FRET.

When excited by 950 nm two-photon excitation the Venus emission of UrVA and UrVV fluorescence emissions in vitro both decayed as a single exponential (τ = 3.1 ± 0.0 ns, mean ± SD, *n* = 3 biologically independent samples, Fig. 2b). These values compared well with the fluorescence lifetime of mVenus excited with 470 nm excitation (τ = 3.03 ± 0.01 ns^43^) or when excited with 950 nm two-photon excitation (τ = 3.1 ± 0.0 ns^21^) indicating the absence of hetero-FRET in these samples. ChVA and ChVV both had a faster average Venus lifetime decay (<τ> = 2.2 ± 0.1 & 2.1 ± 0.1 ns respectively) as determined by amplitude-weighted double exponential curve fitting. Hetero-FRET analysis using these lifetimes indicated an average FRET efficiency (E) for ChVA of 29 ± 3% and for ChVV 32 ± 3%. Assuming an isotropic distribution of mVenus and mCherry dipole orientations in the static averaging regime and using a mVenus to mCherry Förster distance (*R*_0_) of 5.8 nm, these FRET efficiencies correspond to an average mVenus to mCherry distance of 6.4 nm for ChVA and 6.1 nm for ChVV^44^. The fluorescence anisotropy decay of UrVA and ChVA appeared to be identical and were well described using a single exponential decay model suggesting these constructs had no detectable homo-FRET. UrVV and ChVV also appeared to have identical anisotropy decays but had a multiexponential decay with two additional fast decay constants that were well fit using a triple exponential decay model and indicated mVenus to mVenus homo-FRET in these samples (Fig. 2c). The rotational correlation time for a Venus monomer is ~16 ns^43^ and should rotate even slower when attached to other proteins. Since all four of these trimeric FP constructs should have similar rotational correlation times, we analyzed all four decays using global fitting^45,46^ where UrVA and ChVA were fit to a single exponential decay model with a linked decay constant (the rotational correlation time) while UrVV and ChVV were fit to triple exponential decay model comprised of the same linked rotational correlation time constant as well as two additional decay constants to capture the fast anisotropy decay associated with Venus–Venus energy transfer. The rotational correlation time for these constructs was 32.8 ± 0.5 ns (mean ± SD, *n* = 3 biologically independent samples). UrVV had an average fast anisotropy decay constant of 1.8 ± 0.1 ns indicating a homo-FRET transfer rate of 0.278 ns^−1^ corresponding to an estimated mVenus–mVenus distance of 5.3 nm (using a mVenus lifetime of 3.1 ns and a mVenus-to-mVenus *R*_0_ value of 5.2 nm). The average fast anisotropy decay constant of ChVV was statistically indistinguishable from UrVV (1.8 ± 0.2 ns). Thus, these in vitro experiments demonstrate that binary-FRET analysis can differentiate molecules that have no FRET (UrVA), homo-FRET only (UrVV), hetero-FRET only (ChVA), as well as molecules with both homo- and hetero-FRET (ChVV).

Binary-FRET can also be used to image the distribution of steady-state anisotropy and average lifetime values of these control constructs when expressed in living cells (Fig. 2d–g). With these fluorescent constructs, a 128 × 128 pixel parallel and perpendicular polarization image set (used to calculate steady-state anisotropy and average fluorescent lifetime images, see methods), typically required 3–5 min for acquisition. We found pixel anisotropy and lifetime values were uniforms both across individual cells and from cell to cell. In excellent agreement with our in vitro binary-FRET data (Supplementary Table 1), in HEK cells ChVV had a steady-state anisotropy of 0.361 ± 0.005 and an average fluorescent lifetime of 1.98 ± 0.02 ns (mean ± SD), UrVV had an anisotropy of 0.341 ± 0.005 and a lifetime of 2.98 ± 0.02 ns, mChVA had an anisotropy of 0.455 ± 0.004 and a lifetime of 1.94 ± 0.08 ns, and UrVA had an anisotropy of 0.451 ± 0.007 and a lifetime of 2.99 ± 0.02 ns.

### Applying binary-FRET to CaMKII–NR2B interactions

**Fig. 3:**
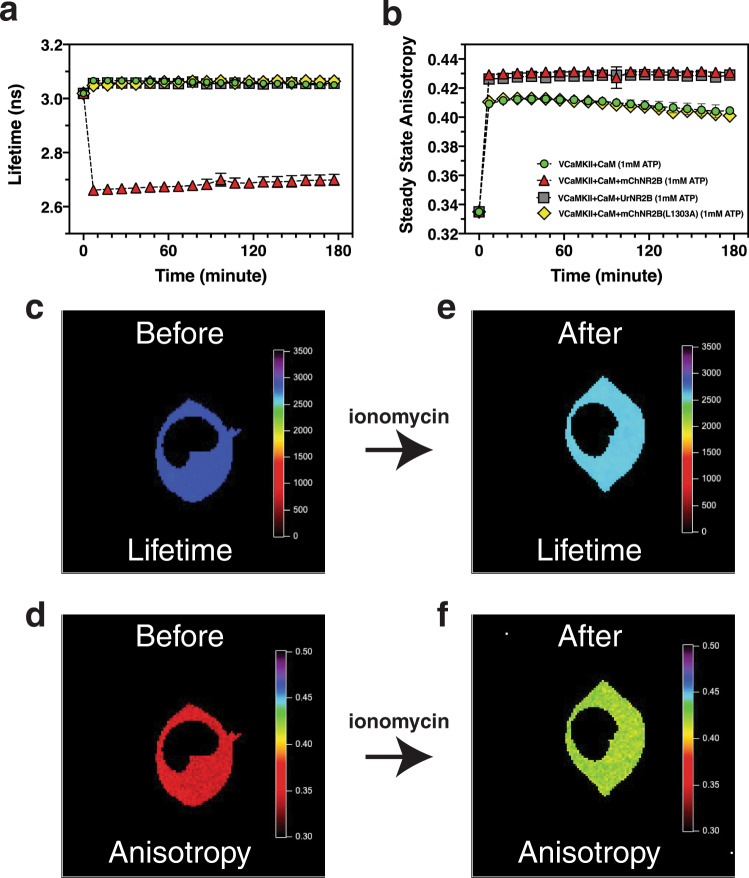
Time course of binary-FRET of CaMKII activation and NR2B association. Changes in the in vitro mVenus fluorescence lifetime (**a**) or steady-state anisotropy (**b**) when V-CaMKII was activated alone (green circles), or in the presence of mCherry-tagged NR2B binding fragment (red triangles), mUranus-tagged NR2B binding fragment (gray squares), or with mCherry-tagged mutant NR2B(L1303A) binding fragment (yellow diamonds). Each point is mean ± SD, *n* = 4 independent samples with two replicates per sample. Representative anisotropy and lifetime decay traces are shown in Supplementary Fig. 2. Live cell binary-FRET lifetime (**c** and **e**) and anisotropy (**d** and **f**) images of HEK cells co-expressing V-CaMKII, mCh-NR2B-binding fragment, and calmodulin before (**c** and **d**) and after (**e** and **f**) activation with ionomycin. Anisotropy color bars ranged from 0.3 (dark red) to 0.5 (dark purple). Lifetime color bars are in picoseconds and range from 0 ps (dark red) to 3500 ps (dark purple). Source data are provided as a Source Data file.

In vitro binary FRET analysis of mVenus fluorescence emitted from catalytic-domain tagged CaMKII*α* (VCaMKII) autoinhibited holoenzyme yielded a fluorescence lifetime of 3.02 ± 0.01 ns (mean ± SD, *n* = 4 biologically independent samples) and a steady-state fluorescence anisotropy value of 0.335 ± 0.002 (Fig. 3a, b at *t* = 0 minutes). Data reduction was used in time-lapse data sets to help visualize dynamic changes in fluorescence lifetime and anisotropy by converting lifetime decay curves into average lifetime values and by converting time-resolved anisotropy decays into steady-state anisotropy values.

Activation of the holoenzyme with 1 mM calcium in the presence of excess (>10 fold) calmodulin (CaM), and 1 mM ATP caused a minor increase in lifetime (3.07 ± 0.01 ns at *t* = 7 min) but triggered a large increase in steady-state anisotropy (that is thought to correspond to a decrease in homo-FRET associated with Venus-tagged catalytic-domain pair separation^25^) that peaked at *t* = 37 min (0.413 ± 0.002) and then slowly decrease (green circles in panels a and b). Activation of the holoenzyme under the same conditions but now in the presence of the T-site ligand mCherry-tagged NR2B-binding fragment (red triangles in panels a and b) resulted in a dramatic drop in lifetime (2.66 ± 0.01 ns at *t* = 7 min) presumably corresponding to NR2B binding to the catalytic domain T-site with a hetero-FRET efficiency of 11.9 ± 0.0%, and now displayed an even larger increase in anisotropy (0.431 ± 0.001 at *t* = 37 min) that remained elevated over 3 h with no apparent decay. We speculate that this further increase in anisotropy is related to a conformational change when NR2B binds to catalytic domain pairs. To verify that the drop in mVenus lifetime was due to hetero-FRET from mVenus to mCherry, we activated V-CaMKII in the presence of Uranus-tagged NR2B binding fragment (gray squares in panels a and b). Substituting mUranus for mCherry on NR2B eliminated its hetero-FRET acceptor and as expected now the mVenus lifetime did not drop upon activation supporting the hypothesis that this lifetime drop was caused by hetero-FRET. Nonetheless, the red and gray anisotropy traces for these constructs were identical, indicating that the exchange of mUranus for mCherry on NR2B did not alter the holoenzyme structure, as reported by mVenus homo-FRET, when NR2B is bound. Finally, we activated V-CaMKII in the presence of a mCherry-tagged mutant of NR2B binding fragment (L1303A) that prevents its binding to CaMKII (yellow diamonds in panels a and b)^18^. Note that the human NR2B mutant L1303A corresponds to the rat NR2B mutant L1298A. As expected, the yellow lifetime and anisotropy traces were indistinguishable from the green traces when NR2B was not present. This supports our hypothesis that the difference between the red (and gray) anisotropy trace and the green (and yellow) trace represents a holoenzyme conformational change that alters mVenus homo-FRET when NR2B binds, while the larger anisotropy change following activation in the green and yellow traces represents a conformational change triggered by holoenzyme activation associated with catalytic-domain pair separation and occurs even in the absence of a functional T-site ligand.

In vitro activation of V-CaMKII*α* in the presence of mCherry-NR2B binding fragment resulted in reciprocal changes in homo- and hetero-FRET, with hetero-FRET increasing while homo-FRET decreased (red traces in Fig. 3a, b). Reciprocal changes were also observed in HEK cells expressing V-CaMKII*α* and mCherry-NR2B when anisotropy and lifetime values were monitored before and 20 min after ionomycin treatment (Fig. 3c–f).

**Fig. 4:**
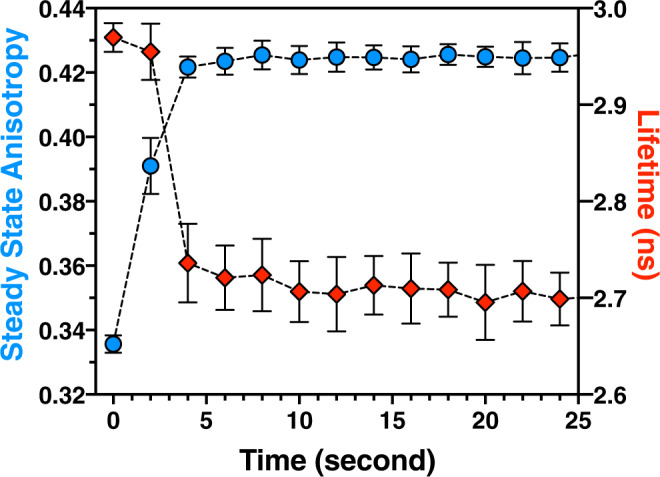
Decrease in V-CaMKII homo-FRET precedes CaMKII–NR2B hetero-FRET. High temporal resolution in vitro binary-FRET was performed using V-CaMKII activated in the presence of mCherry-tagged NR2B binding fragment in a solution containing 5% glycerol. Each point is mean ± SD, *n* = 6 independent samples. Source data are provided as a Source Data file. Representative anisotropy and lifetime decay traces are shown in Supplementary Fig. 3, as are the six independent steady-state anisotropy and average lifetime traces used to calculate the average values shown above.

To test if one of these changes preceded the other, we measured in vitro binary-FRET of V-CaMKII*α* activated with mCherry-NR2B using an increased sampling rate of 0.5 Hz and included 5% glycerol to slow down binding kinetics (Fig. 4). A >50% maximal anisotropy change was observed in the first data point following activation while a statistically significant change in mVenus fluorescence lifetime was only detected in subsequent time points indicating that the change in anisotropy precedes the change in lifetime. This suggests that activation triggers catalytic domain-pair separation, and if a T-site ligand is available, it then can bind to the holoenzyme. This experiment also demonstrates that in vitro binary-FRET can be used to study molecular interactions over a time span ranging from seconds to hours.

### Using binary-FRET to characterize CaMKII mutants

**Fig. 5:**
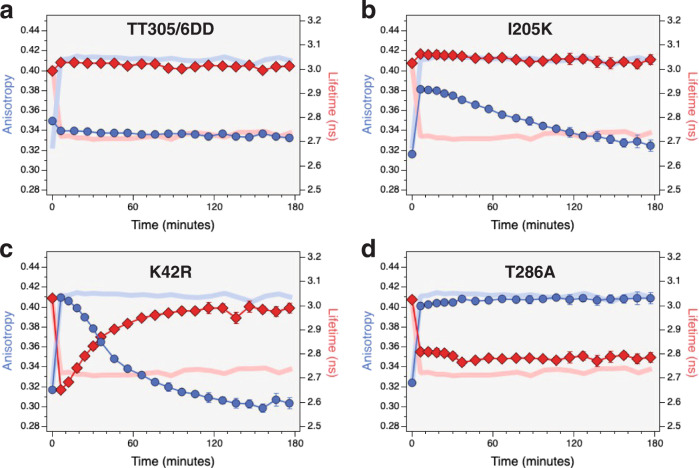
In vitro binary-FRET analysis of mVenus-tagged mutant CaMKII*α* holoenzyme association with mCherry-tagged NR2B binding fragment. The TT305/6DD mutant (**a**), I205K mutant (**b**) K42R mutant (**c**), and T286A mutant (**d**) were activated with excess calcium calmodulin in a buffer containing 1 mM ATP and 10-fold excess mCherry-tagged NR2B-binding fragment. Anisotropy measurements are depicted as blue circles while lifetime measurements are depicted as red diamonds. The anisotropy (solid pink) and lifetime (solid blue) traces of wild-type V-CaMKII’s interaction with mCherry-tagged NR2B binding fragment was added to each panel for comparison. All points are mean ± SD, *n* = 3 independent samples with two replicates per sample. Source data are provided as a Source Data file. Representative anisotropy and lifetime decay traces are shown in Supplementary Fig. 4.

Mutations that alter CaMKII translocation are powerful tools for studying the relationship between holoenzyme structure and NR2B binding. We used binary-FRET analysis to monitor CaMKII*α* catalytic domain separation and subsequent NR2B binding-fragment association using four CaMKII*α* mutants, TT305/6DD, I205K, K42R, and T286A (Fig. 5). Mutation of threonine 305 and 306 to aspartates in the regulatory domain of the kinase are phosphomimic alterations that prevent calcium calmodulin from binding to the holoenzyme and therefore prevents holoenzyme activation^5,18,29^. Binary-FRET analysis confirms that catalytic-domain pair separation, measured as an increase in anisotropy (caused by a decrease in homo-FRET between mVenus-tagged catalytic domains) did not occur with this mutant (Fig. 5a blue circles). Similarly, a decrease in mVenus lifetime, associated with mCherry-tagged NR2B binding-fragment binding was also not observed (red diamonds). For comparison, the anisotropy and lifetime responses of WT-V-CaMKII*α* are shown as solid blue and pink traces, respectively. This experiment demonstrates that calcium-calmodulin binding is required for catalytic-domain separation and subsequent NR2B binding. It also indicates that under our experimental conditions catalytic-domain pairing is stable for at least 3 h.

I205K is a mutation that blocks NR2B binding and activity-dependent holoenzyme translocation to post-synaptic spines^19,29^. Activation with calcium-calmodulin triggered a transient rise in anisotropy (decrease in homo-FRET) but no drop in lifetime (hetero-FRET) (Fig. 5b). The rise in anisotropy was ~2/3 that of wild-type CaMKII*α* (0.381 ± 0.001 and 0.411 ± 0.003, respectively) and then decreased to baseline values over the next three hours. Thus, upon activation catalytic domain pairs separate in the I205K mutant, but without NR2B binding they slowly regain proximity.

Another mutant, K42R^47^, was used to probe the role of ATP in regulating CaMKII*α* interactions with NR2B. This lysine-to-arginine mutant blocks ATP binding in the kinase catalytic site. There are conflicting reports on whether this mutant can translocate to synaptic terminals following activation^5,18^. Like I205K, the K42R mutation also displayed a rapid but transient rise in anisotropy to 0.401 ± 0.001 with a subsequent decay in anisotropy with a slow time constant of ~51 ± 4 min (Fig. 5c). The subsequent drop in anisotropy fell to levels well below those found in the autoinhibited holoenzyme observed at *t* = 0, suggesting that re-associated catalytic domains have a different pairing structure as compared to the autoinhibited holoenzyme. Upon activation the K42R mutant’s lifetime rapidly dropped from 3.03 ± 0.01 to 2.65 ± 0.01 ns, corresponding to a 12.6 ± 0.4% hetero-FRET efficiency. The lifetime subsequently increased almost to starting values with a time constant of ~29 ± 2 min. The different rate constants for anisotropy and lifetime recovery suggest that in the K42R mutant bound NR2B might sterically limit the re-establishment of catalytic domain pairing until they dissociate from the kinase and indicates that the NR2B-binding fragment can transiently bind to this mutant. Transient NR2B binding to the K42R mutant also suggests that ATP might regulate NR2B binding, either structurally, by binding to the catalytic domain, or via subsequent kinase activity. To investigate if T286 autophosphorylation was required for prolonged NR2B binding we next investigated the T286A mutant. Like the K42R mutant, there are also conflicting reports on whether this T286A mutant can translocate to synaptic terminals following activation^5,18,19,29^. Like the wild-type (WT) holoenzyme a rapid and persistent change in the mutant T286A V-CaMKII*α* anisotropy and lifetime was observed upon activation (Fig. 5d). While the increase in anisotropy was almost identical to the change observed in WT CaMKII*α*, the drop in T286A lifetime was slightly attenuated compared to WT (2.72 ± 0.01 and 2.81 ± 0.00 ns, respectively, at *t* = 6 min). Clearly, T286 autophosphorylation is not required for NR2B binding.

### Characterizing the calcium dependence of CaMKII–NR2B association and disassociation

**Fig. 6:**
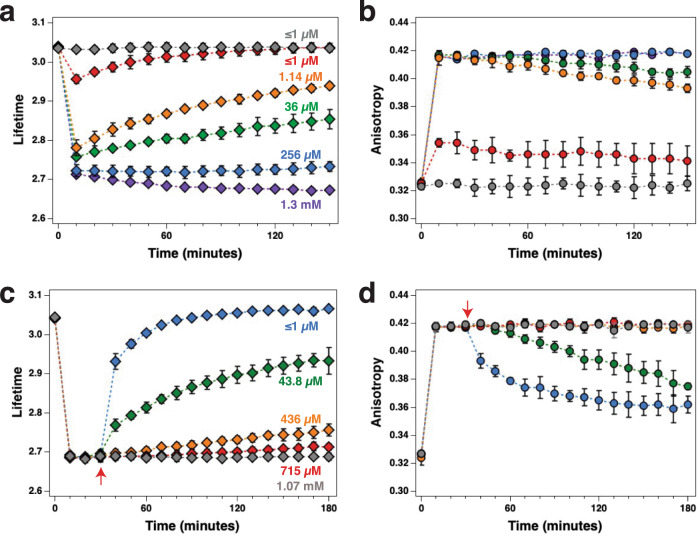
mCherry-NR2B association and disassociation from mVenus-CaMKII*α* as a function of calcium concentration. In vitro Venus lifetime (**a**) and anisotropy (**b**) was measured when 0.2 (gray), 0.5 (red), 0.7 (orange), 1.0 (green), 1.5 (blue), and 3.0 mM (purple) CaCl_2_ was added to our reaction to activate CaMKII. Representative anisotropy and lifetime decay traces depicting the calcium dependence of activation are shown in Supplementary Fig. 5. Following activation of mVenus-CaMKIIα and mCherry-NR2B binding in response to 1.07 mM free calcium, the calcium chelator EGTA was added to test if NR2B binding was reversible. Venus lifetime (**c**) and anisotropy (**d**) was measured when 0.2 (red), 0.5 (orange), 1.0 (green), and 3.0 mM (blue) EGTA was added after *t* = 30 min (red arrows). Representative anisotropy and lifetime decay traces depicting the effects of adding EGTA are shown in Supplementary Fig. 6. Colored text in panels a and c indicate estimated free calcium concentrations. All points are mean ± SD, *n* = 3 independent samples with two replicates per sample. Source data are provided as a Source Data file.

The lifetime traces in Fig. 3a suggests that CaMKII–NR2B binding is persistent over a 3-h period when calcium is maintained at high levels. Intracellular resting *free calcium*, the concentration of the fraction of the total calcium in a solution that is ionized and available to activate biological processes, is typically nano-molar, but can transiently reach concentrations in the hundreds of micro-molar in microdomains near the mouths of open calcium channels^48–50^. It is not known how stable CaMKII–NR2B interactions are under more physiologically relevant calcium concentrations. To investigate the calcium dependence of NR2B binding to CaMKII we monitored the Venus fluorescence lifetime and anisotropy of V-CaMKII*α* when the calcium concentration of the reaction buffer was altered in buffers containing excess calmodulin relative to the V-CaMKII*α* concentration (Fig. 6a, b). CaCl_2_ at 0.2 mM didn’t elicit any change in lifetime or anisotropy (gray traces). In contrast, 0.5 mM CaCl_2_ triggered a transient drop in Venus lifetime and a transient rise in anisotropy (red traces). The lifetime values returned to their initial starting value exponentially with a time constant of 45 ± 8 min while the anisotropy recovered much slower with a time constant of 243 ± 124 min. Using a calcium electrode^51,52^, it was determined that the 0.5 mM CaCl_2_ buffer used corresponded to a free calcium concentration below 1 µM, the lower limit of the linear response of our electrode. 1.14 µM free calcium (0.7 mM CaCl_2_) triggered a larger transient drop in a lifetime and a larger transient rise in anisotropy (orange traces). Now the lifetime recovered with a slower time constant of 154 ± 10 min and the anisotropy recovered with a time constant of 487 ± 66 min. 36 µM free calcium (1 mM CaCl_2_, green traces) also triggered a transient drop in a lifetime and rise in anisotropy with recovery time constants of 315 ± 25 and 885 ± 145 min, respectively. 256 µM free calcium (1.5 mM CaCl_2_, blue traces) triggered a larger drop in lifetime to a value of 2.72 ± 0.01 ns, and a rise in anisotropy to its maximal value of 0.416 ± 0.001. At this high free calcium concentration, the lifetime and anisotropy no longer appeared to return to their initial values. Finally, 1.3 mM free calcium (3 mM CaCl_2_, purple traces) triggered a rapid drop in a lifetime to a value of 2.71 ± 0.01 ns, followed by a much slower further drop to 2.67 ± 0.01 ns at *t* ≥ 130 min. A rise in anisotropy to its maximal value of 0.416 ± 0.002 was also observed with 1.3 mM free calcium. It was notable that the recovery of the lifetimes (red, orange, and green traces) was always significantly faster than the recovery of the corresponding anisotropy signals suggesting that the re-association of catalytic domain pairs was limited by the rate of CaMKII–NR2B disassociation. While it is expected that calcium redistribution among the calmodulin population will influence the kinetics of CaMKII activation, the kinetics we are primarily observing occurs over tens of minutes while calcium–calmodulin equilibrium is thought to occur within seconds^53^.

The transient drop in Venus fluorescence lifetime observed (red, orange, and green traces in panel a) suggests a previously undescribed CaMKII holoenzyme behavior: *T-site binding adaptation*. Following activation with calcium–calmodulin the holoenzyme affinity for NR2B increases rapidly with a higher initial affinity that then attenuates (as a function of the free calcium concentration). It also suggests that CaMKII–NR2B association is reversible. To further investigate the reversibility of NR2B binding the kinase was first activated (1.07 mM free calcium) and 30 min later EGTA was added to chelate the free calcium while monitoring Venus lifetime and anisotropy (Fig. 6c, d). When 0.2 mM EGTA was added the free calcium dropped from 1.07 mM to 715 µM and a slight increase in a lifetime was observed (panel c red trace). No change in the anisotropy was observed (panel d red trace). When 0.5 mM EGTA was added the free calcium dropped to 436 µM and a slightly higher increase in a lifetime was now observed (orange trace). Still, no change in the anisotropy was observed. The addition of 1 mM EGTA dropped the free calcium to 43.8 µM and resulted in a significant rise in a lifetime and now a drop in anisotropy was observed (green traces). The addition of 3 mM EGTA dropped the free calcium to below 1 µM and resulted in a rapid rise in lifetime asymptoting to its initial value (3.07 ± 0.00 at *t* = 3 h), indicating that NR2B binding is fully reversible. A ~60% recovery in anisotropy was observed (blue traces) indicating that under these conditions’ catalytic domain unpairing is not completely reversible, suggesting that other unknown factors can influence catalytic domain re-association.

## Discussion

We present and validate a method for simultaneously monitoring two FRET reactions, one encoded in the fluorescence lifetime of the donor, the second encoded in its anisotropy (Fig. 2). We demonstrate that this approach can be used in vitro for time-lapse measurements allowing the detection of conformational changes and binding reactions with resolutions spanning seconds to hours. Alternatively, binary-FRET can be used in an imaging mode to visualize changes in homo- and hetero-FRET, albeit with reduced temporal resolution. An advantage of binary-FRET is that it utilizes only a small fraction of the available spectral bandwidth. A limitation, however, is that high NA objectives can hinder the separation of anisotropy and lifetime signals^42,54^. Since binary-FRET can potentially measure both CaMKII catalytic domain pair separation and NR2B binding simultaneously, we used it both to test the hypothesis that CaMKII activation triggers a conformational change in the holoenzyme that then allows NR2B binding, as well as to demonstrate the utility of binary-FRET.

Activation of mVenus-CaMKII*α* resulted in a rapid increase in anisotropy (Fig. 3b) interpreted as a decrease in homo-FRET due to catalytic-domain pair separation and is consistent with our previous findings in hippocampal neurons^25^. Catalytic-domain pair separation was observed in both the absence or presence of the mCherry-NR2B binding fragment, but in its absence, the rise in anisotropy was attenuated and subsequently decayed. A full rise in anisotropy was also observed with the Uranus-tagged NR2B, while an attenuated rise with subsequent decay was observed when the mCherry-tagged mutant NR2B_L1303A_-binding fragment was used. Activation of mVenus-CaMKII*α* with mCherry-NR2B resulted in a large drop in Venus lifetime that is interpreted as an increase in hetero-FRET when mCherry-NR2B binds to the holoenzyme (Fig. 3a). Supporting this interpretation, a lifetime drop was not observed when NR2B was tagged with mCherry_Y72C_ (Uranus) or when mCherry-tagged NR2B_L1303A_-binding fragment was used. Together these experiments demonstrate that activation triggers catalytic-domain pair separation, even in the absence of NR2B, and suggests that NR2B binding stabilizes this conformational change. Binary-FRET at 0.5 Hz revealed that catalytic-domain pair separation preceded NR2B binding (Fig. 4). This was observed in every experiment (*n* = 6).

The use of CaMKII*α* mutants revealed additional details of the relationship between catalytic domain separation and NR2B binding (Fig. 5). Since the CaMKII*α*_TT305/6DD_ mutant prevents calmodulin binding and thus activation^5,18,29^ it was not surprising that changes in anisotropy and lifetime were not observed when mVenus-CaMKII*α*_TT305/6DD_ was used (Fig. 5a). A transient and attenuated rise in anisotropy was observed with the CaMKII*α*_I205K_ mutant but a drop in lifetime was no longer observed as compared to wild type holoenzyme (Fig. 5b). The I205K mutant is known to block NR2B binding^19^, and our lifetime data is consistent with this. The transient rise in I205K anisotropy reflects catalytic-domain pair separation, but presumably, in the absence of NR2B binding, unpaired catalytic domains can now re-associate with other catalytic domains. In one study CaMKII*α*_K42R_ failed to translocate^18^ but did in other studies^5,19,29^. A drop in lifetime and a rise in anisotropy were observed using binary-FRET, but these changes were transient indicating that NR2B binding occurs, but then is subsequently released from the holoenzyme (Fig. 5c). Since K42R prevents ATP binding to the kinase^47^, our data suggest that bound ATP is required to maintain NR2B association. One possibility is that bound ATP is needed to allow autophosphorylation at T286. T286 autophosphorylation is known to cause persistent and calcium-independent CaMKII activity. CaMKII*α*_T286A_, which cannot be auto-phosphorylated, also did not translocate to PSDs in one study^18^ but did in other studies^5,19,29^. Using binary-FRET a rapid rise in anisotropy and drop in a lifetime was observed with CaMKII*α*_T286A_ and these changes were persistent over three hours (Fig. 5d) indicating that T286 autophosphorylation is not needed for NR2B binding though the extent of the lifetime drop was attenuated. Presumably, the impaired NR2B binding seen in K42R or T286A can manifest as impaired translocation under some assay conditions.

While we cannot rule out the possibility that time-dependent loss of ATP, uncontrolled dephosphorylation, or proteolysis, during 3-h in vitro experiments, might adversely influence observed binary-FRET measurements, the dramatically different and in most cases reciprocal changes in lifetime and anisotropy observed for V-CaMKII*α* and four mutants (Fig. 5), under identical buffer and incubation conditions, suggests that these potential complications are at best minor concerns.

Since binary-FRET revealed that: 1. Catalytic-domain pair separation occurred upon CaMKII*α* activation, even in the absence of a T-site ligand, 2. Unpairing always preceded ligand binding, and 3. T-site ligand binding was never observed without catalytic domains pair separation, we conclude that calcium–calmodulin triggers a transition from a compact auto-inhibited state where catalytic domains are paired and T-sites are hidden, to an intermediate state with unpaired catalytic domains with exposed T-sites. We hypothesize that exposed T-sites are now free to support three general types of activation-triggered molecular interactions, 1. Reassociation of catalytic domains into pairs^27^, 2. CaMKII holoenzyme aggregation^29,55^, and 3. T-site ligand binding^5,18,19,29^.

Upon activation, the holoenzyme initially displayed a higher affinity for NR2B, and with time affinity decreased under steady-state sub-maximal calcium conditions (Fig. 6a). The decay constant for NR2B dissociation changed with calcium, the higher the calcium the slower the dissociation. Understanding the mechanistic underpinnings of the relationship between free calcium and NR2B binding, and subsequent dissociation is challenging since calmodulin has four calcium binding sites with a range of affinities that interact cooperatively to bind calcium^56,57^. While calcium-occupancy of these sites regulates CaMKII activation, further complexity might arise from having multiple Ca^2+^/calmodulin-binding sites in the holoenzyme that interact cooperatively to regulate kinase activity^27^, as well as from multiple T-sites in the holoenzyme that also interact cooperatively^27^. Kinase activity, such as autophosphorylation, apparently is not needed for this adaptation, as biphasic NR2B binding was also observed with the K42R mutant (Fig. 5c). Biphasic NR2B binding also indicates that NR2B binding is at least partially reversible. Complete reversibility was confirmed by chelating calcium after NR2B binding (Fig. 6c). While the physiological function of adaptation of T-site ligand binding affinity is not known, we suggest that adaptation might be part of a mechanism that allows CaMKII holoenzyme to sense and respond to transient calcium gradients, specifically it could support directed holoenzyme translocation toward the mouths of NMDA receptor channels via a series of directionally biased T-site binding and dissociation steps. While the mechanism of this adaptation remains to be determined, calcium-triggered long-lived and dynamic CaMKII conformational changes that appear to be required for subsequent NR2B binding are consistent with a role in forming a biological eligibility trace.

## Methods

### Plasmid information

Trimeric control plasmids: mCherry–mVenus–mVenus (ChVV), mCherry_Y72C_–mVenus–mVenus (UrVV), mCherry–mVenus–mVenus_Y67C_ (ChVA) and mCherry_Y72C_–mVenus–mVenus_Y67C_ (UrVA), were derived from the trimeric plasmid mVenus–mVenus–mVenus (VVV)^25^. The initial mVenus was replaced with mCherry by restriction enzyme cloning from mCherry-C1 to form mCherry–mVenus–mVenus precursor. The mCherry-C1 vector was initially produced by replacement of the EGFP open reading frame of pEGFP-C1 (Takara/Clontech) with the mCherry open reading frame from pmCherry-NLS (pmCherry-NLS was a gift from Martin Offterdinger (Addgene plasmid # 39319; http://n2t.net/addgene:39319; RRID: Addgene_39319)). In-Fusion Cloning (Takara) was used to exchange the last mVenus open-reading frame with a codon-optimized mVenus (mVenusHP^58^). At this point, the three fluorescent protein coding segments no longer had repetitive sequences and allowed for traditional PCR-based site-directed mutagenesis (Q5® Site-Directed Mutagenesis (NEB)) to generate the various chromophore null mutant trimers listed above.

The mVenus-mCaMKII*α* (V-CaMKII) plasmid was generated by amplifying mCaMKIIa (NM_177407.4) from mouse whole brain cDNA (Clontech #639400, Mouse Brain Marathon®-Ready cDNA). The open reading frame was cloned into the mVenus-C1 vector by restriction enzyme-based cloning. The mVenus-C1 vector (Addgene #27794) was created by swapping the mVenus open reading frame into the eGFP-C1 vector (Clontech #6084-1: GBacc# U55763). The T286A, TT305/6, I205K, and K42R mutants were modified from wildtype V-CaMKII with QuickChange Mutagenesis using the following primers:

T286A: Sense 5′-ATGCACAGACAGGAG**GCC**GTGGACTGCCTGA-3’

Antisense 5′-TCAGGCAGTCCAC**GGC**CTCCTGTCTGTGCAT-3’

TT305/306DD: Sense 5′-GGAGCCATCCTC**GACGAC**ATGCTGGCCACCAGG-3′

Antisense 5′-CCTGGTGGCCAGCAT**GTCGTC**GAGGATGGCTCC-3′

I205K: Sense 5′-TCATCCTGTAT**AAG**TTGCTGGTTGG-3’

Antisense 5′-CCAACCAGCAA**CTT**ATACAGGATGA-3’

K42R: Sense 5′-AGTATGCTGCC**AGG**ATTATCAACAC-3’

Antisense 5′-GTGTTGATAAT**CCT**GGCAGCATACT-3’

Fluorescently labeled T-site ligand mCherry-NR2B was generated by In-Fusion cloning of nucleotides 3793–3960 (amino acids 1265–1320) of human NR2B (GRIN2B: NM_000834) into the mCherry-C1 vector. A Flag epitope was included between the mCherry and NR2B fragment. This construct was used as an In-Fusion template for the insertion of mCherry-NR2B into the pRSETB (Thermo) bacterial expression vector downstream, and in frame with, the vector 6His tag. Uranus-NR2B (mCherry_Y72C_-NR2B) and the T-site null mCherry-NR2B mutant (L1303A) were generated in the above bacterial mCherry-NR2B expression construct by Q5® Site-Directed Mutagenesis.

6His-Venus, 6His-Venus_Y67C_ (Amber), 6His-mCherry, and 6His-mCherry_Y72C_ (Uranus) (see Supplementary Fig. 1), were all constructed in the pRSETB vector by In-Fusion Cloning using constructs listed above as templates.

Human calmodulin (GB accession number NM_ 001743.4) cDNA was previously subcloned into pCI Mammalian Expression Vector (Promega, Madison, WI) using XhoI and SmaI restriction sites using primer pair sense 50-ATTCTCGAGCAGCATGGCTGACCAACTGACTG-30 and antisense 50-GGGGATATCTCACTTCGCTGTCATCATTTGTAC-30^27^.

### Bacterial protein production and purification

Bacterial expression vectors were transformed into BL21(DE3) competent *E. coli* (C2527H: NEB), plated on selection plates and incubated overnight at 37 °C. single colonies were harvested and grown in 60 mL of LB media overnight at 37 °C. Cultures were induced with 1 mM IPTG (final) and shaken at 180 RPM for 6–7 h at room temperature (~22 °C). Cultures were pelleted by centrifugation; the culture media was removed, and pellets were stored at −30 °C until purification.

For protein purification, biomass (pellet from 60 mL culture) was resuspended in 4 mL of 50 mM Tris, 200 mM NaCl, pH 8.0 with Halt Protease Inhibitor (Thermo), lysed by sonication and centrifuged at 100,000 × *g* for 30 min at 10 °C. The supernatant was filtered through a 0.22 mm syringe filter (Millipore). Filtered supernatant was purified on an NGC Quest 10 Plus Chromatography System (Bio-Rad). First, a 1 mL EconoFit Nuvia IMAC Column (Bio-Rad) was used for the affinity chromatography purification step, where 50 mM Tris, 200 mM NaCl, 20 mM Imidazole, pH 8.0 was used as the wash buffer, next a wash buffer with 250 mM Imidazole was used for elution. Eluted protein-containing fractions were concentrated, if necessary, in an Amicon Ultra 2 mL Concentrator (Millipore). Proteins were further purified by size-exclusion chromatography on an ENrich SEC 650, 10 × 300 mm column (Bio-Rad), using the same Tris, NaCl buffer, but without Imidazole. Purified proteins were concentrated as needed in Amicon Ultra 2 mL concentrators (Millipore).

### Time-lapse in vitro binary-FRET

Time-lapse binary-FRET experiments were implemented on a home-built two-photon microscope (see Supplementary Fig. 7a) based on our previous auto-FPFA design^59^. Specifically, an 80 MHz, 70-fs Ti:Sapphire laser (MaiTai eHP, Spectra-Physics) operated at 950 nm was employed for two-photon excitation. After passing a Glan laser (GL-10B, Thorlabs) where the ordinary ray was removed, the excitation beam was filtered and expanded (K310, Thorlabs). It was then directed to an optical group consisting of a near-IR half-wave plate (AHWP10M-980, Thorlabs) and a linear polarizer (LPVIS100-MP2, 10^8^:1 extinction ratio at 950 nm, Thorlabs) so that the excitation power and polarization at the sample can be adjusted. Next, the laser beam was guided through a multiphoton long-pass dichroic beam splitter (FF665-Di02-25 × 36, Semrock) to an air microscope objective (Zeiss ×40 0.9 NA, with back aperture, slightly overfilled) which focuses the beam to a diffraction-limited spot (~0.5 μm in diameter). Fluorescence emitted from excited Venus fluorophores was collected by the same microscope objective and reflected (by the dichroic beam splitter) to a group consisting of a multiphoton short-pass filter (FF01-680/SP-25, Semrock) to block residual near-IR photons and a bandpass filter (FF01-525/50-25, Semrock) to remove photons emitted from mCherry fluorophores. The Venus emission then was directed to a polarizing beam splitter cube (PBS251, Thorlabs) augmented with two orthogonally oriented linear polarizers (LPVISA100-MP2, Thorlabs) where parallel and perpendicular emitted photons were separated, and each signal was detected by its own dedicated hybrid detector (HPM-100-40, Becker & Hickl). Signals associated with parallel and perpendicular detected photons from the hybrid detectors were combined by a router (HRT-41, Becker & Hickl) and then sent to an SPC-130EM TCSPC card (Becker & Hickl) to construct parallel and perpendicular fluorescence lifetime decays, respectively. For synchronization with excitation pulses, a small fraction of the excitation beam was extracted and focused onto a fast photodiode (DET10N, Thorlabs) whose output signal was sent to the synchronization channel of the SPC-130EM card.

Homogenates from cells expressing fluorescent protein-tagged constructs were pipetted into a glass-bottom 96-well microplate (Greiner Bio-One) which was attached to an *XY* motorized stage (HLD117, Prior Scientific). The stage was anchored via ∅1.5” damped posts (Thorlabs) to minimized mechanical vibrations. Samples were scanned over the Zeiss microscope objective which was inversely mounted on a post-mountable focus block (MGZ30, Thorlabs). A motorized microscope focus controller (MFC1, Thorlabs) was attached to the MGZ30 fine adjustment knob to provide computerized *Z*-axis adjustments.

The SPC-130EM card was controlled by SPCM software (Becker & Hickl, Version 9.67, 32 bit) running in FIFO mode for data acquisition, storage, and calculation of time-resolved fluorescence from micro-time data. In addition, a custom LabView (National Instruments, Version 2015) program was developed for computerized manipulation of the *XY* and *Z* motorized stages.

Two-photon excitation power, at 950 nm, was maintained at ~9.6 mW (at the sample plane) to prevent bleaching during acquisition (~60 s per measurement). For time-lapse binary-FRET, measurement at each point was an average of at least two repeats. Even though mVenus fluorescence was typically observed over a 3-h time course, mVenus fluorescence excitation occurred only over a small fraction of this time (0.092) to avoid photobleaching. All samples were prepared and measured at room temperature.

### Live-cell imaging binary-FRET

Imaging binary-FRET in living cells can be achieved utilizing the principles of laser scanning microscopy as depicted in Supplementary Fig. 7b. In our experimental setup, an 80 MHz, 70-fs Ti:Sapphire laser (MaiTai, eHP, Spectra-Physics) was tuned to 950 nm to provide two-photon excitation of the Venus FP. A Glan laser (GL10-B, Thorlabs) was placed in the excitation beam path to remove the ordinary ray. The laser beam was then passed through a combination of a near-IR half-wave plate (AHWP10M-980, Thorlabs) and a linear polarizer (LPVIS100-MP2, 10^8^:1 extinction ratio at 950 nm, Thorlabs) so that the excitation power and polarization at the sample can be manipulated. Next, the beam was directed through a long-pass multiphoton dichroic beam splitter (FF665-Di02-25×36, Semrock) to a 2-dimensional galvo scanner (LSKGG4, Thorlabs) attached with a telecentric scan lens (SL50-CLS2, Thorlabs). The scan lens was positioned next to the right-side port of a Zeiss Axio Observer Z1 microscope so that the distance between it and the microscope’s intermediate image plane matches the lens’s working distance. This arrangement yielded an infinity-corrected optical system (see Thorlabs application note at: https://www.thorlabs.com/newgrouppage9.cfm?objectgroup_id=2910&pn=SL50-CLS2). After entering the microscope (via the right-side port), the excitation beam was focused to a diffraction-limited spot (~0.5 μm in diameter) in the sample plane using a Zeiss, ×40, 0.9NA objective. Fluorescence emitted from the observation volume was collected by the same microscope objective and passed through the right-side port and 2D galvo scanner. After the scanner, the emission was reflected by the dichroic beam splitter and then filtered by a multiphoton short-pass filter (FF01-680/SP-25, Semrock) to block residual near-IR photons and a bandpass filter (FF01-525/50-25, Semrock) to remove photons emitted from mCherry fluorophores. The Venus emission was next guided to a polarizing beam splitter cube (PBS251, Thorlabs) where parallel and perpendicular emitted photons are separated, and each signal was detected by its own dedicated hybrid detector (HPM-100-40, Becker & Hickl). Note that the cube was augmented with two orthogonally oriented linear polarizers (LPVISA100-MP2, Thorlabs) to enhance its polarization extinction ratio. Signals associated with parallel and perpendicular detected photons from the hybrid detectors, after passing a router (HRT-41, Becker & Hickl), as well as spatial information (*X* and *Y* positions) of the excitation beam obtained from the 2D galvo scanner were combined, and sent to SPC-150N TCSPC card (Becker & Hickl) to construct parallel and perpendicular fluorescence lifetime decay images, respectively. For synchronization, a small fraction of the excitation beam was extracted and focused onto a fast photodiode (DET10N, Thorlabs) whose output signal was also sent to the SPC-150N card.

SPCM software (version 9.86, 64 bit, Becker & Hickl) was used for live-cell imaging data acquisition, storage and for creating 128 × 128 pixel parallel and perpendicular fluorescence lifetime decay images. These images were transferred to SPCImage (version 8.5, Becker & Hickl) for further analysis (see binary-FRET image data analysis and processing below). Cells transfected with constructs of interest were plated on glass-bottom Petri dishes (P35GC-1.5-14-C, MatTek) at very low densities so that they were well separated. Only cells with dim fluorescence were selected for imaging to avoid intermolecular non-specific FRET. Two-photon excitation power was maintained around ~5 mW (after the objective) to prevent bleaching during the acquisition (typically between 3 and 5 min).

### Cell culture, transfection and homogenate preparation

HEK cells (tsa201 human cell line, sku 96121229) were purchased from Sigma Aldrich. Cell culture, transfection, and homogenate preparation were performed as previously described^59^, HEK cells were cultured as a monolayer in a T-75 Flask in a humidified incubator containing 9% CO_2_ in air at 37 °C in DMEM (1X) + GlutaMAXTM-1 media containing d-glucose, sodium pyruvate, and 10% fetal bovine serum. A day prior to binary-FRET measurement, cells were suspended using TrypLE Express and washed with DPBS (without calcium and magnesium). Plasmid DNA encoding Venus-tagged constructs (typically 1 μg/250,000 cells) were transfected into the cells using electroporation. Transfected cells were plated on 60 mm culture dishes and incubated overnight. On the following day, cells were harvested and lysed using a passive lysis buffer. Homogenates were centrifuged at 100,000 × *g* for 30 min to remove membranes and particulate matter. Supernatants were diluted for binary-FRET to yield a photon count rate between ~20,000 cps (>25× the dark count rate) and <100,000 cps. Clarified homogenates (200 μl) were then loaded into 96-well glass bottom plates and measured by in vitro binary-FRET. For the validation of binary-FRET, supernatants of tissue culture homogenates expressing ChVV, UrVV, ChVA, and UrVA were diluted in DPBS buffer (contains no calcium chloride and magnesium chloride, Gibco by Life Technologies) and measured in a 96-well glass bottom microplate (Greiner Bio-One). For the experiments related to CaMKII–NR2B interaction, prior to a time-lapse binary-FRET measurement, an activation buffer was prepared, consisting of 1× kinase buffer (10× KinEASE^TM^ buffer, EMD Millipore), 1 mM DTT (Sigma Aldrich), 1 mM CaCl_2_ (Sigma Aldrich), 1 mM ATP (Thermo Scientific), 1 mM MgCl_2_ (Sigma Aldrich), and 5 µM Calmodulin (CaM, EMD Millipore) in the final reaction. Supernatants of tissue culture homogenates expressing mVenus-tagged CaMKII*α* constructs (holoenzyme concentration varies from 15 to 20 nM) were incubated in the above activation buffer either with or without 10 µM purified mCherry-tagged NR2B (see above). The final reaction mixture was typically 200 µl which was pipetted into a well of a 96-well glass-bottom microplate (Greiner Bio-One). In some experiments, CaCl_2_ in the buffer was replaced by 1 mM EGTA (AG Scientific) to chelate calcium. For rapid kinetic measurements, 5% glycerol w/v (Invitrogen) was added to a sample buffer containing mVenus-tagged CaMKII*α* homogenates (15–20 nM) and purified mCherry-tagged NR2B (10 µM). This buffer (180 µl) was loaded into our sample chamber (a 96-well glass-bottom plate) and a time-lapse series of binary-FRET measurements were initiated at a sampling rate of 0.5 Hz. Kinase activation was initiated by pipetting 20 µl of activation buffer into our sample chamber while the data acquisition was in progress. For imaging binary-FRET, Ionomycin (Sigma Aldrich) was added to DPBS buffer (containing CaCl_2_ and MgCl_2_, Gibco by Life Technologies) at a final concentration of 2 μM to trigger Ca^2+^/CaM activation inside cells co-expressing V-CaMKII, mCh-NR2B-binding fragment, and calmodulin.

### In-vitro data analysis

To observe changes over time in homo-FRET between Venus fluorophores, steady-state anisotropy was estimated based on parallel and perpendicular fluorescence decays using the following equation:

$$R=\frac{\sum {I}_{\parallel }\left(t\right)-G\cdot \sum {I}_{\perp }(t)}{\sum {I}_{\parallel }\left(t\right)+2\cdot G\cdot \sum {I}_{\perp }(t)}$$

where *I*_II_(*t*) and *I*_⊥_(*t*) are time-dependent fluorescence intensity decays of parallel and perpendicular channels (dark noise subtracted) respectively, and the instrument correction factor *G* for our microscope had a value of 1.07 as determined by calibration using fluorescein (NIST tracible standard, Life Technologies) tail fitting. The value of *G* was measured every two weeks while conducting experiments and was found to be stable over several months.

To monitor changes in hetero-FRET between Venus and mCherry fluorophores over time, the fluorescence lifetime decays (Eq. (4)) were fit using a double exponential decay model, and the amplitude weighted *average lifetime* was calculated using the following equation:

$$\bar{\tau }=\frac{{a}_{1}.{\tau }_{1}+{a}_{2}.{\tau }_{2}}{{a}_{1}+{a}_{2}}$$

where *a*_1_ and *a*_2_ are the amplitudes of the two exponential decay components and *τ*_1_ and *τ*_2_ are the two-time constants of the individual decay components.

### Statistics and reproducibility

All calculations, including standard and global fitting of time-resolved fluorescence intensity, and time-resolved anisotropy were performed through an analysis tool developed based on IGOR Pro software (WaveMetrics Inc., Version 9.00). Calculated steady-state anisotropy and/or average mVenus lifetime values were transferred to GraphPad Prism 7 for time-dependent plots as well as statistical calculation of means and standard deviations (SD). No statistical method was used to predetermine the sample size. No data were excluded from the analyses. The experiments were not randomized. The Investigators were not blinded to allocation during experiments and outcome assessment.

### Estimating the distance between fluorophores in a dimer by homo-FRET

Assuming an isotropic population of rapidly moving fluorophores in solution, the distance, *R*, between two identical fluorophores can be estimated from fluorescence anisotropy and lifetime data based on the following equation:

$$R=\frac{{R}_{0}} { \root {6} \of {\frac{\tau }{2\phi }}}$$

where τ is the fluorescent lifetime of the FRET donor, *R*_0_ is the Förster distance of the homo-FRET pair, and *ϕ* is the fast anisotropy decay constant related to homo-FRET in a dimer anisotropy decay trace. The single-step homo-FRET energy transfer rate of a dimer, *ω*, can be calculated from *ϕ* using the following equation:

$$\omega=\frac{1}{2\phi }$$

### Binary-FRET image data analysis and processing

A schematic depicting the binary-FRET image processing data pipeline is shown in Supplementary Fig. 8. Raw parallel and perpendicular fluorescence lifetime decay images, acquired using SPCM software, were transferred to SPCImage software for further processing. SPCImage was used to calculate parallel and perpendicular polarization intensity images, ${I}_{\parallel }\left(x,\,y\right)$ and ${I}_{\perp }\left(x,\,y\right),$ the integrated parallel or perpendicular photon count at each pixel, where *x* and *y* indicate image pixel coordinates. Then ${I}_{\parallel }\left(x,\,y\right)$ and ${I}_{\perp }\left(x,\,y\right)$ images were used to calculate a steady-state anisotropy image, $R\left(x,\,y\right)$ using the following equation:

7 $$R\left(x,\,y\right)=\frac{{I}_{\parallel }\left(x,\,y\right)-G\cdot {I}_{\perp }\left(x,\,y\right)}{{I}_{\parallel }\left(x,\,y\right)+2\cdot G\cdot {I}_{\perp }\left(x,\,y\right)}$$

The instrument correction factor, *G*, for our imaging setup had a value of 0.922 as determined by calibration using fluorescein tail fitting.

The SCPImage was also used to fit parallel and perpendicular fluorescence lifetime decay images using a triple exponential decay model to fit the decay trace at each individual pixel location. This was used to generate parallel and perpendicular amplitude weighted average lifetime images, ${\bar{\tau }}_{\parallel }\left(x,\,y\right)$ and ${\bar{\tau }}_{\perp }\left(x,\,y\right)$, as well as peak amplitude images, ${A}_{\parallel }\left(x,\,y\right)$ and ${A}_{\perp }\left(x,\,y\right)$. A triple exponential fit was used to generate the best estimates of amplitude weighted average lifetime, and peak amplitude at each pixel. These four images could then be used to generate an amplitude-weighted average fluorescence lifetime image, $\bar{\tau }\left(x,\,y\right)$, using the following equation:

8 $$\bar{\tau }\left(x,\,y\right)=\frac{{A}_{\parallel }\left(x,\,y\right)\cdot {\bar{\tau }}_{\parallel }\left(x,\,y\right)+2\cdot G\cdot {A}_{\perp }\left(x,\,y\right)\cdot {\bar{\tau }}_{\perp }\left(x,\,y\right)}{{A}_{\parallel }\left(x,\,y\right)+2\cdot G\cdot {A}_{\perp }\left(x,\,y\right)}$$

This equation was derived as follows considering for simplicity only the values measured at a single pixel. Since the total fluorescence intensity following a laser excitation pulse decays with time as

$$I\left(t\right)={I}_{\parallel }\left(t\right)+2\cdot G\cdot {I}_{\perp }\left(t\right)$$

the above equation can be rewritten to incorporate parallel and perpendicular decay amplitudes and decay constants:

$$A{\cdot {{{{{\rm{e}}}}}}}^{-\frac{t}{\bar{\tau }}}={A}_{\parallel }\cdot {{{{{{\rm{e}}}}}}}^{-\frac{t}{{\bar{\tau }}_{\parallel }}}+2\cdot G\cdot {A}_{\perp }\cdot {{{{{{\rm{e}}}}}}}^{-\frac{t}{{\bar{\tau }}_{\perp }}}$$

Thus, at *t* = 0, $A={A}_{\parallel }+2\cdot G\cdot {A}_{\perp }$. Next to find the amplitude-weighted average fluorescence lifetime, $\bar{\tau }$, we integrate the above equation:

$${\int }_{\!\!\!0}^{{{\infty }}}A{\cdot {{{{{\rm{e}}}}}}}^{-\frac{t}{\bar{\tau }}}{{{{{{\rm{d}}}}}}t}={\int }_{\!\!\!0}^{{{\infty }}}{A}_{\parallel }\cdot {{{{{{\rm{e}}}}}}}^{-\frac{t}{{\bar{\tau }}_{\parallel }}}{{{{{{\rm{d}}}}}}t}+2\cdot G\cdot {\int }_{\!\!\!0}^{{{\infty }}}{A}_{\perp }\cdot {{{{{{\rm{e}}}}}}}^{-\frac{t}{{\bar{\tau }}_{\perp }}}{{{{{{\rm{d}}}}}}t}$$

Solving this equation, one can find that the value of $\bar{\tau }$ at each pixel is

$$\bar{\tau }=\frac{{A}_{\parallel }\cdot {\bar{\tau }}_{\parallel }+2\cdot G\cdot {A}_{\perp }\cdot {\bar{\tau }}_{\perp }}{{A}_{\parallel }+2\cdot G\cdot {A}_{\perp }}$$

Custom image-processing software programmed in IGOR-Pro (version 9.00, WaveMetrics) was used to calculate *R*(*x*, *y*) and $\bar{\tau }\left(x,\,y\right)$ images as outlined above using ${I}_{\parallel }\left(x,\,y\right)$ & ${I}_{\perp }\left(x,\,y\right)$, ${\bar{\tau }}_{\parallel }\left(x,\,y\right)$ & ${\bar{\tau }}_{\parallel }\left(x,\,y\right)$, and ${A}_{\parallel }\left(x,\,y\right)$ & ${A}_{\perp }\left(x,\,y\right)$ images generated using SCPImage software. IGOR-Pro first read and stored these six images. Next, a mask image was generated for the data set using IGOR-Pro’s built-in Threshold function applied to the parallel intensity image ${I}_{\parallel }\left(x,\,y\right)$. The threshold function used an automated iterative algorithm to set a threshold value that determines which pixels have sufficient signal for further processing. The mask image then was used during subsequent calculations of *R*(*x*, *y*) and $\bar{\tau }\left(x,\,y\right)$ images to remove pixels with aberrant values due to a low photon count. The custom software used Eq. (7) to generate an anisotropy image from ${I}_{\parallel }\left(x,\,y\right)$ & ${I}_{\perp }\left(x,\,y\right)$ images and Eq. (8) to generate an average fluorescence lifetime image using ${\bar{\tau }}_{\parallel }\left(x,\,y\right)$ & ${\bar{\tau }}_{\parallel }\left(x,\,y\right)$, and ${A}_{\parallel }\left(x,\,y\right)$ & ${A}_{\perp }\left(x,\,y\right)$ images as inputs. The custom IGOR-Pro software can also work in a batch-mode to input and process ${I}_{\parallel }\left(x,\,y\right)$ & ${I}_{\perp }\left(x,\,y\right)$, ${\bar{\tau }}_{\parallel }\left(x,\,y\right)$ & ${\bar{\tau }}_{\parallel }\left(x,\,y\right)$, and ${A}_{\parallel }\left(x,\,y\right)$ & ${A}_{\perp }\left(x,\,y\right)$ images from time-lapse data sets to generate a bundle of steady-state anisotropy image *R*(*x, y*) and amplitude weighted average lifetime image $\bar{\tau }\left(x,\,y\right)$ automatically.

### Free Ca^2+^ measurements

Free calcium measurements were estimated using the calcium electrode technique described by Tsien^51,52^. A calcium electrode (KWIKCAL-2, World Precision Instruments) connected to a pH meter (model 370, Orion) was used to estimate free calcium concentration in replicate buffers of those used to study the calcium dependence of CaMKII–NR2B association and disassociation (Fig. 6). Two sets of buffers (1 mL final volume per point) were prepared. The first set, used for studying NR2B association, consisted of 6 points while the second set, used for NR2B disassociation, consisted of 5. In the association set, each point contained 1× kinase buffer, 1 mM DTT, 1 mM ATP, 1 mM MgCl_2_, 1 mM EGTA, 5 µM calmodulin, and 20 nM CaMKII*α* holoenzyme (in a tissue-culture homogenate prepared from HEK cells expressing mVenus-tagged CaMKII*α*). CaCl_2_ was added to these buffers to a final concentration of 0.2, 0.5, 0.7, 1, 1.5, and 3 mM. The disassociation set, contained 1× kinase buffer, 1 mM DTT, 1 mM ATP, 1 mM MgCl_2_, 1 mM CaCl_2_, 5 µM calmodulin, and 20 nM CaMKIIα holoenzyme. In this set EGTA was then titrated to 0, 0.2, 0.5, 1, and 3 mM EGTA. Electrode calibration measurements were performed using a set of eight calcium buffers (CALBUF-1, World Precision Instruments) to yield a calibration curve relating electrode potential (mV) as a function of the known free calcium concentration of these buffers. This calibration revealed that the Log of the free calcium concentration was a linear function of electrode potential for buffers having ≥1 µM free calcium, thus setting the lower limit of detectable calcium using this assay. This linear relationship was then used to estimate free calcium concentrations in our association and disassociation buffer points using experimentally derived electrode potential readings.

### Reporting summary

Further information on research design is available in the Nature Research Reporting Summary linked to this article.

## Supplementary information

Supplementary Information

Reporting Summary

## Data Availability

All data used in this study are presented in this article, and in Supplementary Information. Source data are provided with this paper. Due to the large number and size of raw Becker & Hickl TCSPC (time correlated single photon counting) data files associated with this study, these files will be made available within a month from the corresponding author upon request.

## Source data

Source Data

## Code availability

The custom Igor-Pro code used in this study are available on https://github.com/ssvogel/binaryFRET.
