# Supplementary material for: Binary-FRET reveals transient excited-state structure associated with activity-dependent CaMKII - NR2B binding and adaptation
Source: Nat Commun. 2022 Oct 25;13:6335. doi: 10.1038/s41467-022-33795-8 (PMC9596428; doi:10.1038/s41467-022-33795-8)
Supplement: Supplementary file 1 — Supplementary Information [file 41467_2022_33795_MOESM1_ESM.pdf]

Supplementary Information

Binary-FRET reveals transient excited-state structure associated with activity-dependent CaMKII - NR2B binding and adaptation.

Tuan A. Nguyen[1], Henry L. Puhl III[1], Kirk Hines[1], Daniel J. Liput[2], Steven S. Vogel[1]*

[1]Laboratory of Biophotonics and Quantum Biology, NIAAA, NIH.
[2] Laboratory of Molecular Physiology, NIAAA, NIH.
[*]Corresponding Author (stevevog@mail.nih.gov)

Contents:

Supplementary Fig. 1 *Characterization of mAmber and mUranus mutants of mVenus and mCherry.*

Supplementary Table 1 *Comparison of in vitro and cell imaging binary FRET data acquired for control constructs for experiments shown in figure 2.*

Supplementary Fig. 2 *Representative anisotropy and lifetime decay traces from data depicted in figure 3.*

Supplementary Fig. 3 *Anisotropy and lifetime traces from data depicted in figure 4*

Supplementary Fig. 4 *Representative anisotropy and lifetime decay traces from data depicted in figure 5.*

Supplementary Fig. 5 *Representative anisotropy and lifetime decay traces from data depicted in figure 6a&b*

Supplementary Fig. 6 *Representative anisotropy and lifetime decay traces from data depicted in figure 6c&d.*

Supplementary Fig. 7 *Schematics for implementing (**a**) in-vitro binary-FRET photometry, and (**b**) live-cell binary-FRET imaging.*

Supplementary Fig. 8 *Schematics depicting the binary-FRET image processing data pipeline.*

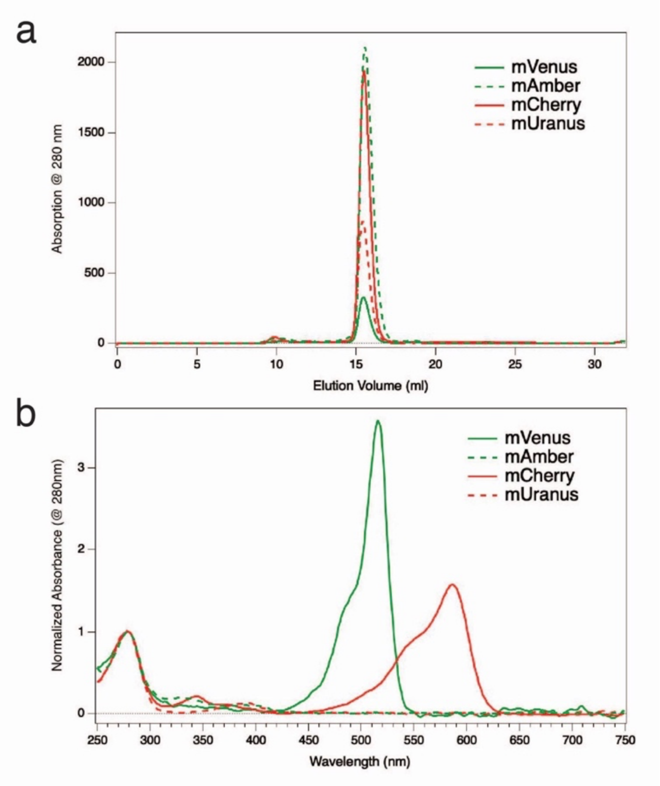

Supplementary Fig. 1 *Characterization of mAmber and mUranus mutants of mVenus and mCherry. 6His-mVenus (solid green traces), 6His-mVenus$_{Y67C}$ (mAmber; dashed green traces), 6His-mCherry (solid red traces) and 6His-mCherry$_{Y72C}$ (mUranus; dashed red traces) were purified by affinity chromatography as described in the Methods and their hydrodynamic volume/ apparent molecular mass was characterized by size exclusion chromatography (**Panel a**). mVenus had an apparent molecular mass of 28.8 kDa, mAmber was 26.8 kDa, mCherry was 28.1 kDa, and mUranus was 29.4 kDa. Since all four fluorescent proteins fell in the range of 27-29 kDa, it is unlikely that the single point mutations in mAmber or in mUranus significantly altered their three-dimensional structure. All four proteins were purified by size exclusion chromatography and their absorbance was measured and normalized to their 280 nm absorption peak (**Panel b**). Note that the tyrosine to cysteine mutations in the mVenus and mCherry chromophores (to create mAmber and mUranus respectively) eliminated all appreciable absorbance above 420 nm, thus mAmber cannot serve as a homo-FRET acceptor for mVenus energy transfer and mUranus cannot serve as a hetero-FRET acceptor for mVenus energy transfer.*

Binary-FRET Control Construct	Hetero FRET	Homo FRET	In vitro Lifetime <τ>	In vitro Anisotropy (steady state)	Cell Imaging Lifetime <τ>	Cell Imaging Anisotropy (steady state)
**ChVV**	✓	✓	2.1 ± 0.1 ns	0.358±0.001	2.0±0.0 ns	0.361±0.005
**UrVV**	X	✓	3.1 ± 0.0 ns	0.344±0.001	3.0±0.0 ns	0.341±0.005
**ChVA**	✓	X	2.2 ± 0.1 ns	0.466±0.000	1.9±0.1 ns	0.455±0.004
**UrVA**	X	X	3.1 ± 0.0 ns	0.461±0.001	3.0±0.0 ns	0.451±0.007

Supplementary Table 1 *Comparison of in vitro and cell imaging binary FRET data acquired for control constructs for experiments shown in figure 2. Values are mean ± SD.*

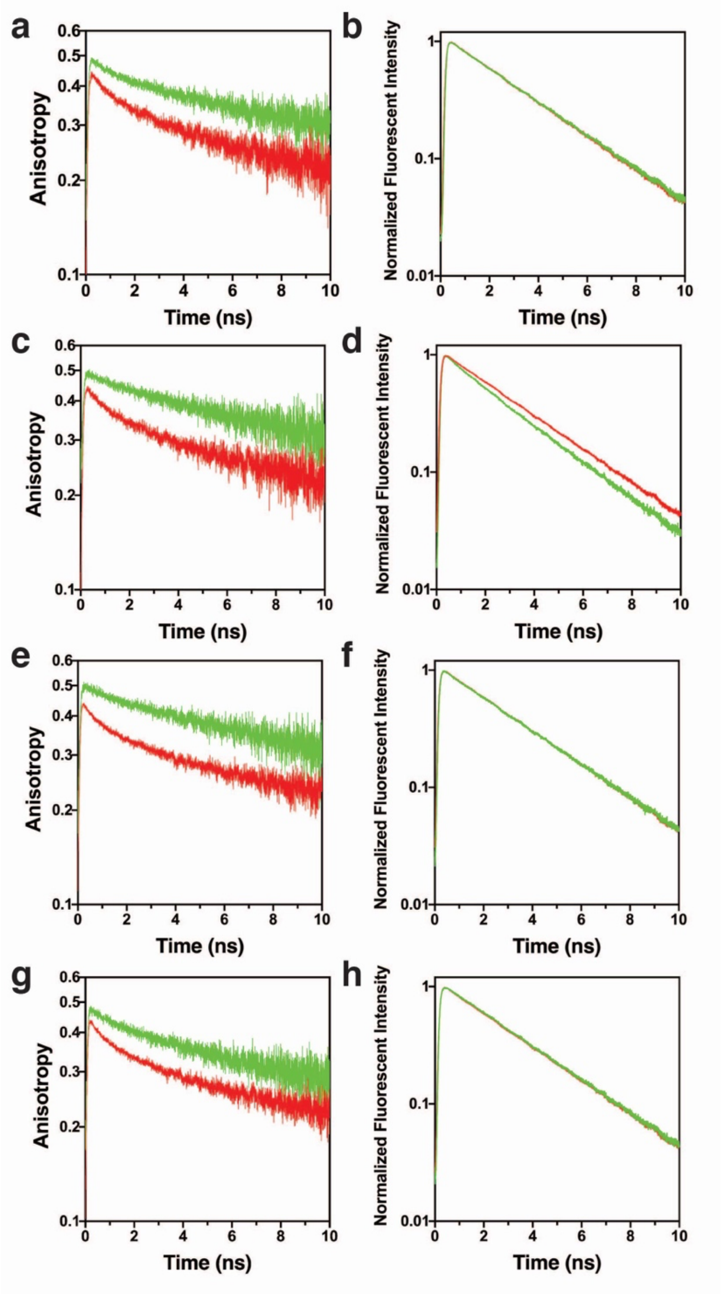

Supplementary Fig. 2 *Representative anisotropy and lifetime decay traces from data depicted in figure 3. Panels **a** & **b** depict representative traces for V-CaMKIIα with calmodulin at 1 mM ATP, **c** & **d** depict representative traces for V-CaMKIIα with calmodulin and mChNR2B at 1 mM ATP, **e** & **f** depict representative traces for V-CaMKIIα with calmodulin and UrNR2B at 1 mM ATP, **g** & **h** depict representative traces for V-CaMKIIα with calmodulin and mChNR2B(L1303A) at 1 mM ATP. Panels **a, c, e,** & **f** show representative fluorescence anisotropy decay traces, while **b, d, f,** & **h** show representative fluorescence lifetime decay traces. **Red** traces are at t=0 before CaMKII activation and **green** traces are at t=177 minutes after activation.*

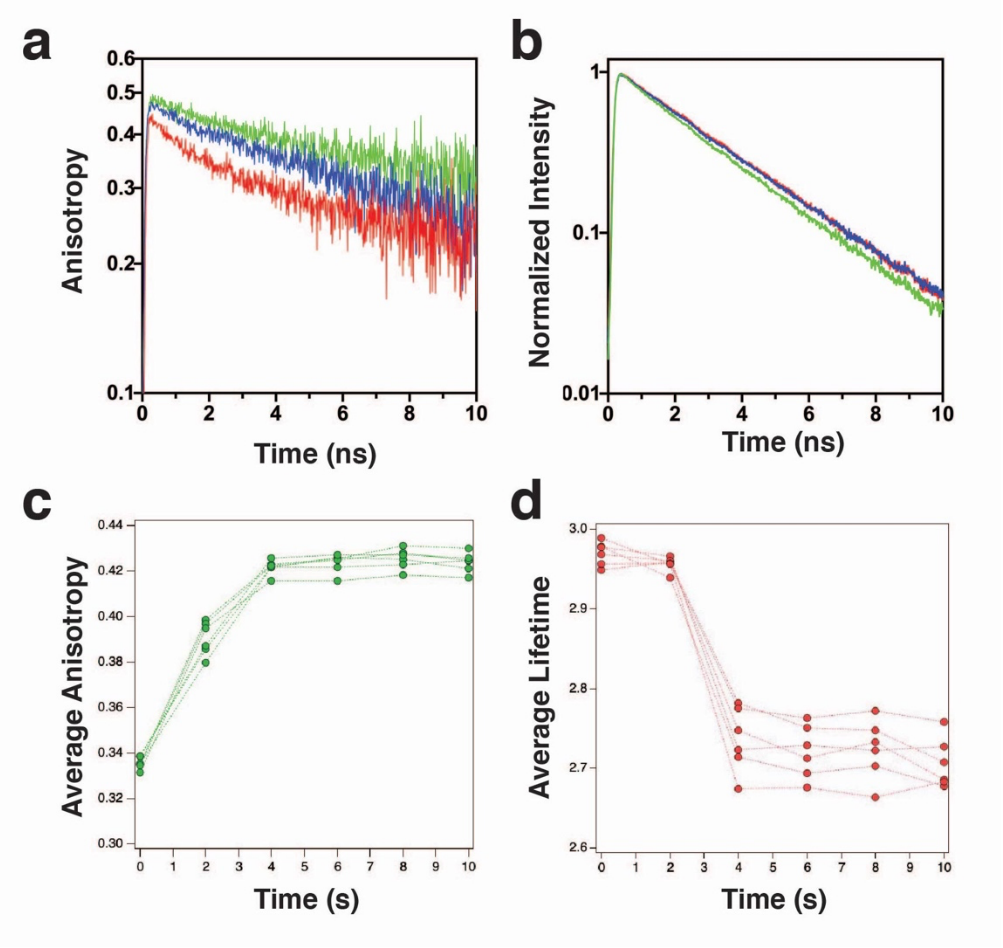

Supplementary Fig. 3 *Anisotropy and lifetime traces from data depicted in figure 4. Panels **a** & **b** depict average (n=6) in vitro anisotropy (**a**) and lifetime (**b**) decay traces for V-CaMKIIα with calmodulin and mChNR2B at 1 mM ATP. **Red** traces are at t=0 before CaMKII activation, **blue** traces are at t=2 seconds after activation, and **green** traces are at t=4 seconds after activation. The time-course of individual anisotropy (**c**) and lifetime (**d**) values of the six replicate in vitro reactions used to calculate the average values presented in figure 4.*

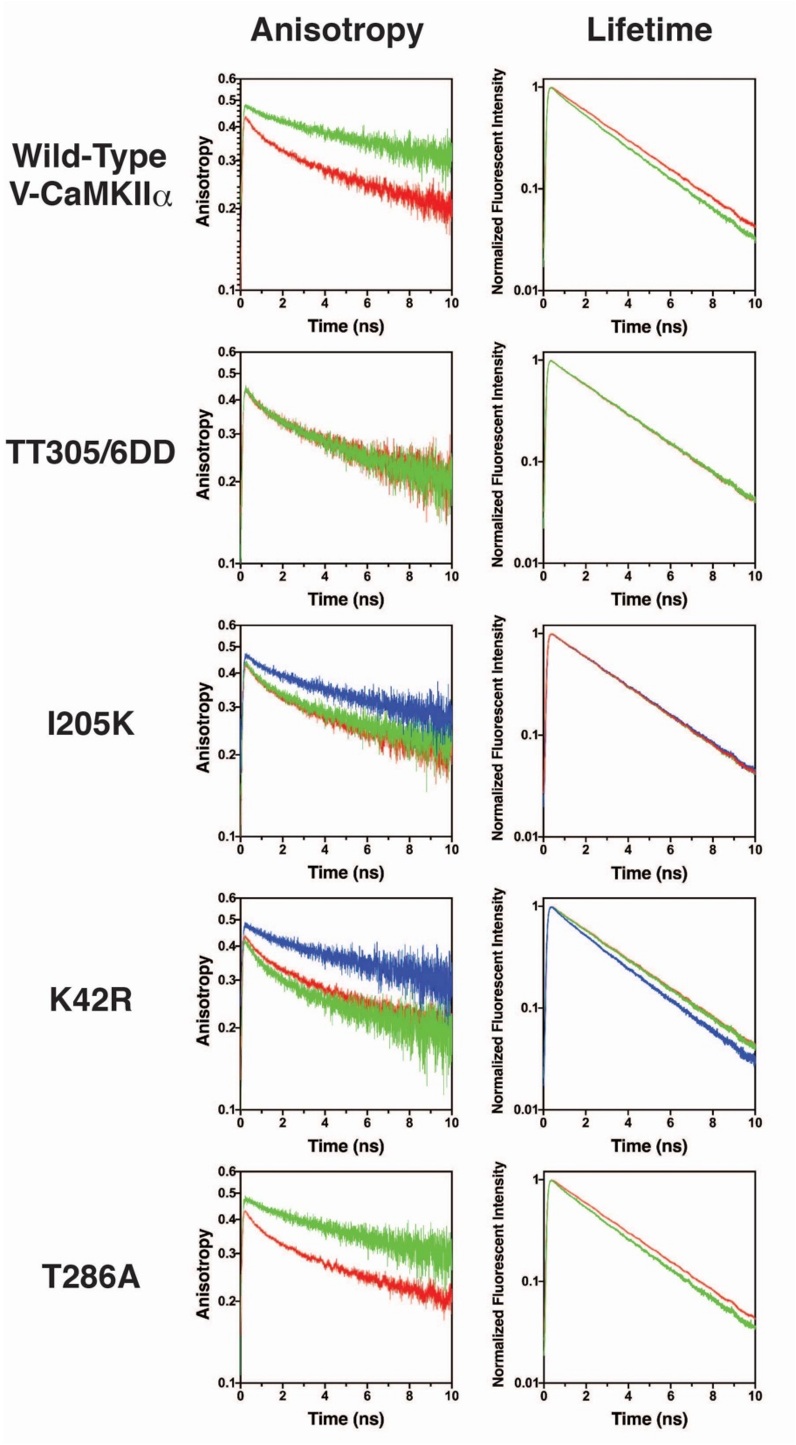

Supplementary Fig. 4 *Representative anisotropy and lifetime decay traces from data depicted in figure 5.* ***Red*** *traces are at t=0 before CaMKII activation,* ***blue*** *traces (shown for I205K and K42R) are at t=6 minutes after activation, and* ***green*** *traces are at t=176 minutes after activation.*

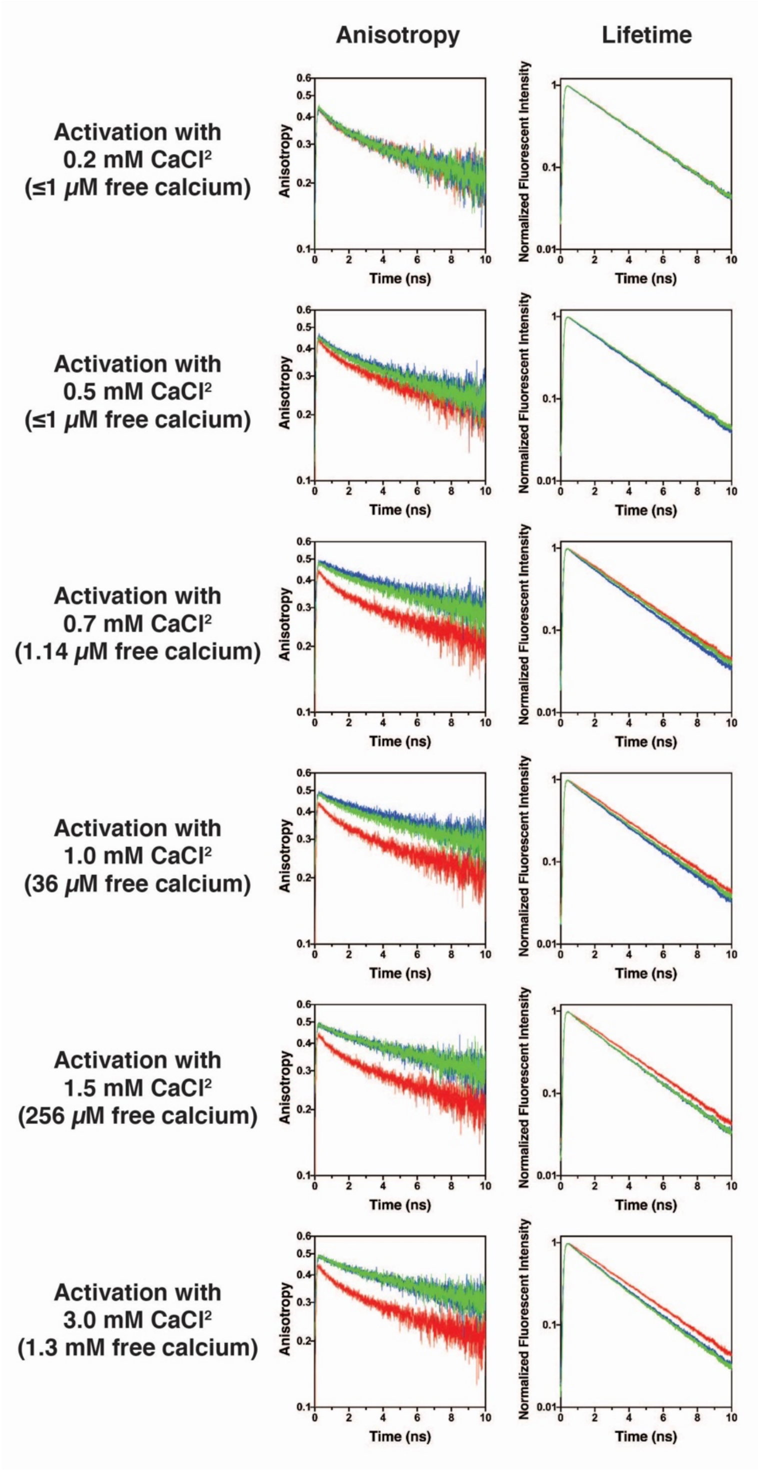

Supplementary Fig. 5 *Representative anisotropy and lifetime decay traces from data depicted in figure 6a&b.* ***Red*** *traces are at t=0 minutes (before the addition of calcium),* ***blue*** *traces are at t=10 minutes after the addition of calcium, and* ***green*** *traces are at t=150 minutes after the addition of calcium.*

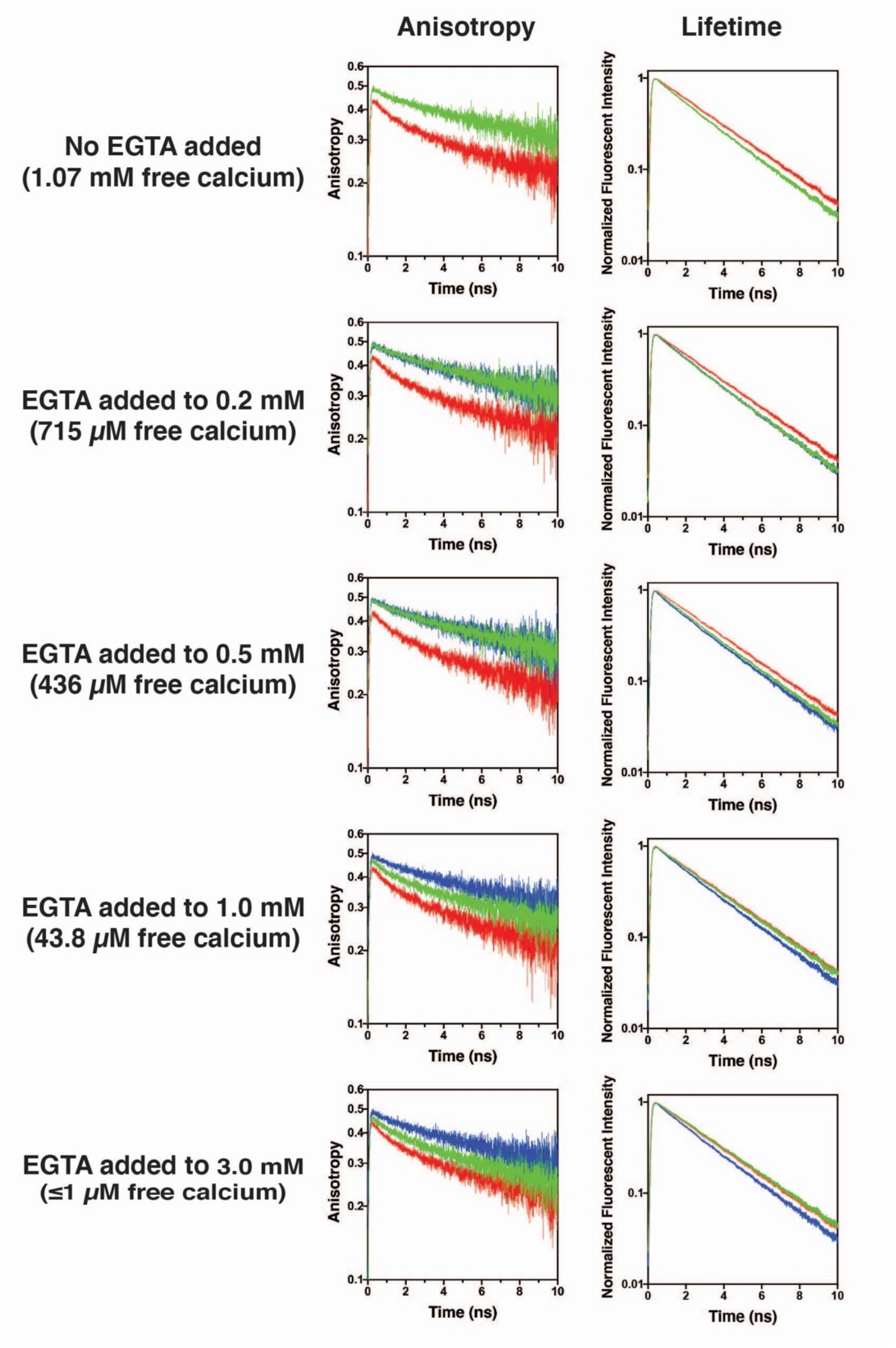

Supplementary Fig. 6 *Representative anisotropy and lifetime decay traces from data depicted in figure 6c&d.* ***Red*** *traces are at t=0 minutes (before activation),* ***blue*** *traces are at t=30 minutes, immediately before the addition of EGTA, and* ***green*** *traces are at t=150 minutes, approximately 120 minutes after the addition of EGTA. Note: Blue traces are not shown in the "no EGTA added" graphs.*

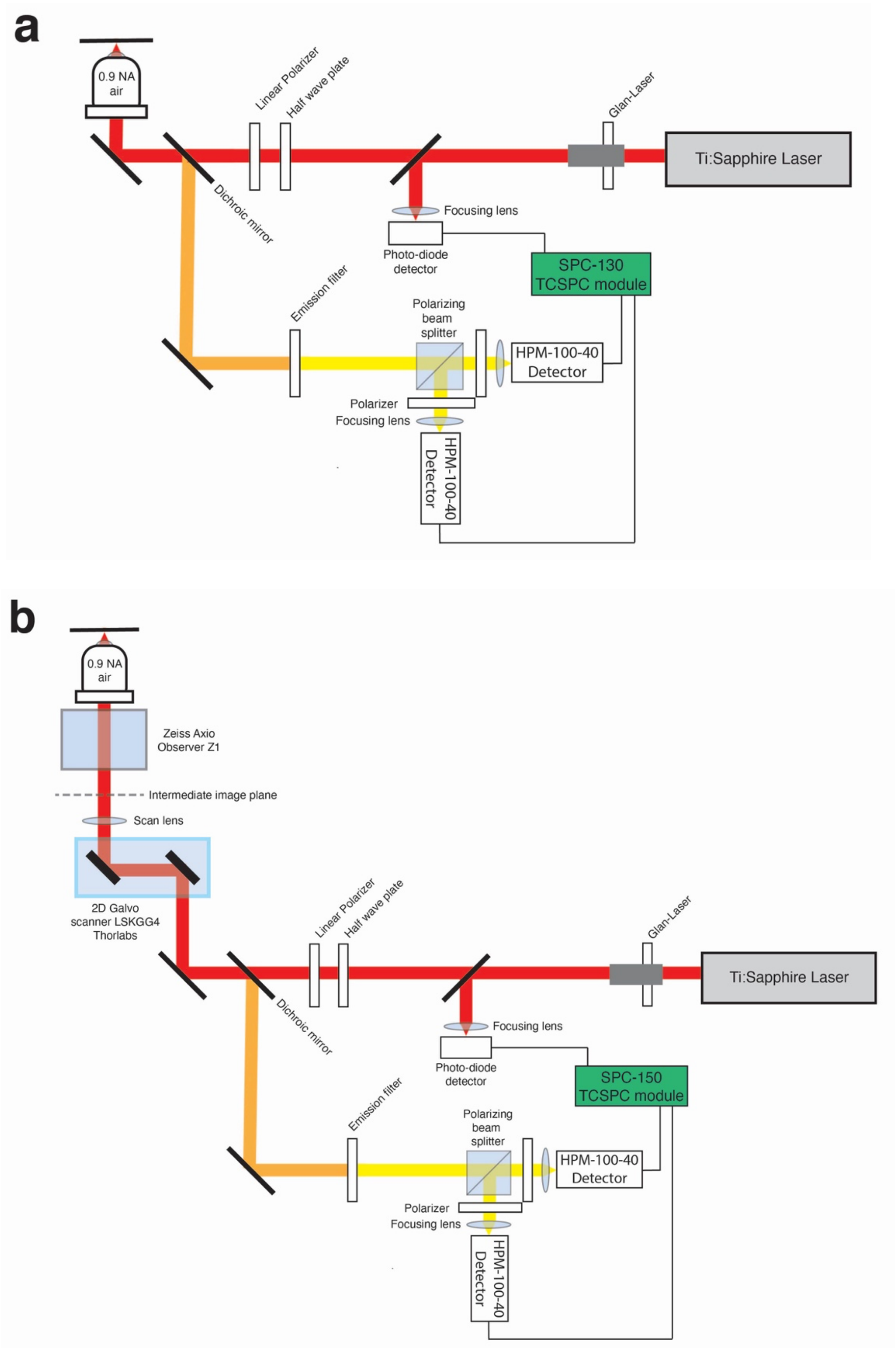

Supplementary Fig. 7 *Schematics for implementing (**a**) in-vitro binary-FRET photometry, and (**b**) live-cell binary-FRET imaging.*

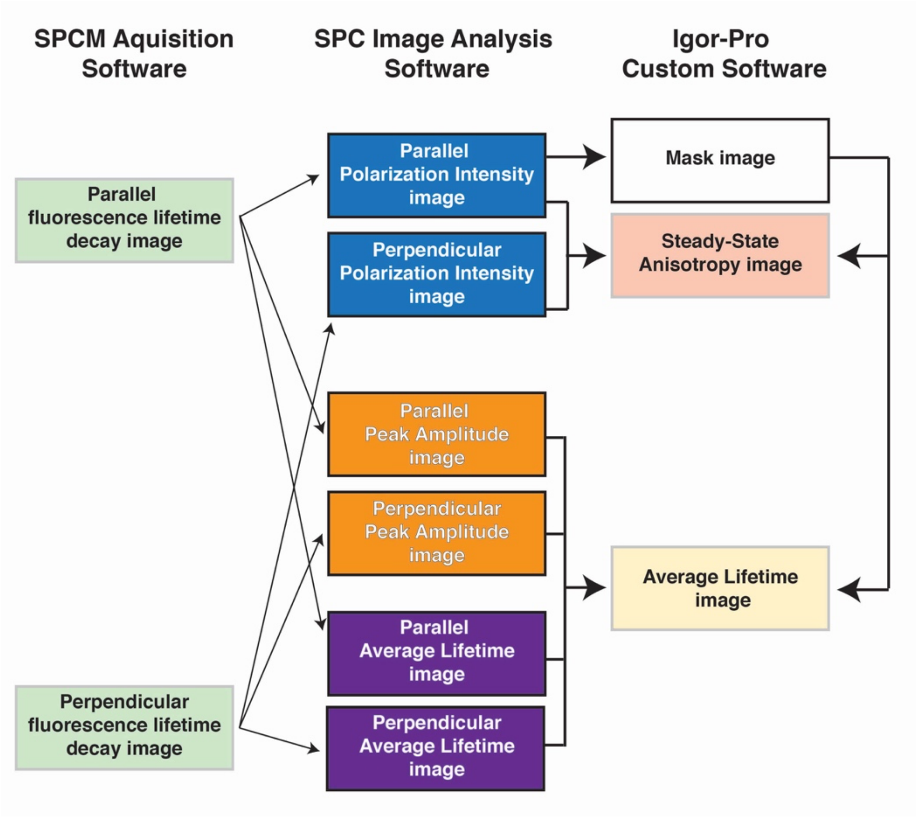

Supplementary Fig. 8 *Schematics depicting the binary-FRET image processing data pipeline. SPCM acquisition software is used to acquire a pair of parallel and perpendicular fluorescence lifetime decay images from the instrumentation depicted in* supplementary *fig. 7b (green boxes). This pair of images are next transferred to SPCImage analysis software to generate three pairs of parallel and perpendicular polarization images of Intensity (blue boxes, Peak Amplitude (orange boxes), and Average Lifetime (purple boxes). These six images are next transferred to custom Igor-Pro software where the pair of parallel & perpendicular polarization intensity images (blue boxes) are used to calculate a steady-state anisotropy image (pink box), and the parallel & perpendicular peak amplitude images (orange boxes) and average lifetime images (purple boxes) are used to calculate an average lifetime image (yellow box). Additionally, a mask image (white box) is generated by applying an automated iterative threshold function to the parallel polarization intensity image. This mask image is then used to mask anomalous pixels (in both the steady-state anisotropy image and the average lifetime image) that are derived from pixel data sets with restrictively low photon count values.*
