# Supplementary material for: Binary-FRET reveals transient excited-state structure associated with activity-dependent CaMKII - NR2B binding and adaptation
Source: Nat Commun. 2022 Oct 25;13:6335. doi: 10.1038/s41467-022-33795-8 (PMC9596428; doi:10.1038/s41467-022-33795-8)
Supplement: Supplementary file 2 — Reporting Summary [file 41467_2022_33795_MOESM2_ESM.pdf]

nature portfolio

Corresponding author(s): Steven Vogel

Last updated by author(s): Sep 27, 2022

Reporting Summary

Nature Portfolio wishes to improve the reproducibility of the work that we publish. This form provides structure for consistency and transparency in reporting. For further information on Nature Portfolio policies, see our Editorial Policies and the Editorial Policy Checklist.

Statistics

For all statistical analyses, confirm that the following items are present in the figure legend, table legend, main text, or Methods section.

n/a	Confirmed	
☐	☒	The exact sample size (n) for each experimental group/condition, given as a discrete number and unit of measurement
☐	☒	A statement on whether measurements were taken from distinct samples or whether the same sample was measured repeatedly
☒	☐	The statistical test(s) used AND whether they are one- or two-sided<br>*Only common tests should be described solely by name; describe more complex techniques in the Methods section.*
☒	☐	A description of all covariates tested
☒	☐	A description of any assumptions or corrections, such as tests of normality and adjustment for multiple comparisons
☐	☒	A full description of the statistical parameters including central tendency (e.g. means) or other basic estimates (e.g. regression coefficient) AND variation (e.g. standard deviation) or associated estimates of uncertainty (e.g. confidence intervals)
☒	☐	For null hypothesis testing, the test statistic (e.g. F, t, r) with confidence intervals, effect sizes, degrees of freedom and P value noted<br>*Give P values as exact values whenever suitable.*
☒	☐	For Bayesian analysis, information on the choice of priors and Markov chain Monte Carlo settings
☒	☐	For hierarchical and complex designs, identification of the appropriate level for tests and full reporting of outcomes
☒	☐	Estimates of effect sizes (e.g. Cohen's d, Pearson's r), indicating how they were calculated

Our web collection on statistics for biologists contains articles on many of the points above.

Software and code

Policy information about availability of computer code

Data collection	SPCM version 9.67 for Windows, 32 bit from Becker & Hickl GmbH was used for in vitro binary-FRET, SPCM version 9.86 for Windows, 64 bit from Becker & Hickl GmbH was used for live cell imaging binary-FRET, LabView full development system 2015, service pack 1,32 bit, for Windows, also FPFA v4.vi software for LabView available at GitHub https://github.com/ssvogel/binaryFRET/
Data analysis	Igor Pro 9.0 for Mac OS, GraphPad Prism 8.0.2 for Mac OS, also Homo and Hetero-FRET fifo.pxp file and SSA-FLIM Images.pxp files for Igor Pro available at https://github.com/ssvogel/binaryFRET

For manuscripts utilizing custom algorithms or software that are central to the research but not yet described in published literature, software must be made available to editors and reviewers. We strongly encourage code deposition in a community repository (e.g. GitHub). See the Nature Portfolio guidelines for submitting code & software for further information.

Data

Policy information about availability of data

All manuscripts must include a data availability statement. This statement should provide the following information, where applicable:

- Accession codes, unique identifiers, or web links for publicly available datasets
- A description of any restrictions on data availability
- For clinical datasets or third party data, please ensure that the statement adheres to our policy

All data used in this study are presented in this article, and in Supplementary Information.

Raw Becker & Hickl TCSPC (time correlated single photon counting) data files will be made available from the corresponding author upon reasonable request.

Human research participants

Policy information about studies involving human research participants and Sex and Gender in Research.

Reporting on sex and gender	n/a
Population characteristics	n/a
Recruitment	n/a
Ethics oversight	n/a

Note that full information on the approval of the study protocol must also be provided in the manuscript.

Field-specific reporting

Please select the one below that is the best fit for your research. If you are not sure, read the appropriate sections before making your selection.

[x] Life sciences [ ] Behavioural & social sciences [ ] Ecological, evolutionary & environmental sciences

For a reference copy of the document with all sections, see nature.com/documents/nr-reporting-summary-flat.pdf

Life sciences study design

All studies must disclose on these points even when the disclosure is negative.

Sample size	all experiments were at least n=3
Data exclusions	no data was excluded
Replication	all experiments are included in the study
Randomization	not randomized
Blinding	not performed

Reporting for specific materials, systems and methods

We require information from authors about some types of materials, experimental systems and methods used in many studies. Here, indicate whether each material, system or method listed is relevant to your study. If you are not sure if a list item applies to your research, read the appropriate section before selecting a response.

Materials & experimental systems

n/a	Involved in the study	
x		Antibodies
	x	Eukaryotic cell lines
x		Palaeontology and archaeology
x		Animals and other organisms
x		Clinical data
x		Dual use research of concern

Methods

n/a	Involved in the study	
x		ChIP-seq
x		Flow cytometry
x		MRI-based neuroimaging

Eukaryotic cell lines

Policy information about cell lines and Sex and Gender in Research

Cell line source(s)	tsa201cell line human embryonic kidney, SV40 transformed purchased from Sigma-Aldrich
Authentication	none performed
Mycoplasma contamination	none tested
Commonly misidentified lines (See ICLAC register)	none found on list
